# Inferring causal genomic alterations in breast cancer using gene expression data

**DOI:** 10.1186/1752-0509-5-121

**Published:** 2011-08-01

**Authors:** Linh M Tran, Bin Zhang, Zhan Zhang, Chunsheng Zhang, Tao Xie, John R Lamb, Hongyue Dai, Eric E Schadt, Jun Zhu

**Affiliations:** 1 Sage Bionetworks, Seattle, WA 98109, USA; 2 Merck Research Laboratories, Merck & Co., Inc., 33 Avenue Louis Pasteur, Boston, MA 02115, USA; 3 Pacific Biosciences, 1505 Adams Drive, Menlo Park, California 94025, USA

**Keywords:** breast cancer, copy number variation, gene regulatory networks, oncogenes

## Abstract

**Background:**

One of the primary objectives in cancer research is to identify causal genomic alterations, such as somatic copy number variation (CNV) and somatic mutations, during tumor development. Many valuable studies lack genomic data to detect CNV; therefore, methods that are able to infer CNVs from gene expression data would help maximize the value of these studies.

**Results:**

We developed a framework for identifying recurrent regions of CNV and distinguishing the cancer driver genes from the passenger genes in the regions. By inferring CNV regions across many datasets we were able to identify 109 recurrent amplified/deleted CNV regions. Many of these regions are enriched for genes involved in many important processes associated with tumorigenesis and cancer progression. Genes in these recurrent CNV regions were then examined in the context of gene regulatory networks to prioritize putative cancer driver genes. The cancer driver genes uncovered by the framework include not only well-known oncogenes but also a number of novel cancer susceptibility genes validated via siRNA experiments.

**Conclusions:**

To our knowledge, this is the first effort to systematically identify and validate drivers for expression based CNV regions in breast cancer. The framework where the wavelet analysis of copy number alteration based on expression coupled with the gene regulatory network analysis, provides a blueprint for leveraging genomic data to identify key regulatory components and gene targets. This integrative approach can be applied to many other large-scale gene expression studies and other novel types of cancer data such as next-generation sequencing based expression (RNA-Seq) as well as CNV data.

## Background

Tumors arise from the activation of oncogenes along with the inactivation of tumor suppressor genes via somatic gene mutations or copy number variation (CNV). Identification of the genetic/genomic changes that drive biological processes associated with cancer onset or progression assists in the development of therapeutics targeting the affected proteins or their downstream consequences[1-4]. Although extensive gene expression studies have been conducted for identifying tumor signature genes associated with poor outcome[5,6], the reproducibility of these signatures is low[7,8], posing a major challenge for identifying the causal genetic/genomic variations.

Genome-wide DNA copy number variation (CNV) has been increasingly used for identifying biomarkers and targets in cancer research[9-11]. Unfortunately, the CGH, SNP genotype, and DNA sequencing data typically used to detect CNV are not available for many published, large-scale studies. Therefore, the development of methods to infer CNV from non-genetic data collected in these studies would serve to enhance their scientific value. Strong correlations between CNV and gene expression have been observed[11,12] and initially suggested the possibility of detecting CNV directly from gene expression data. Recently, the Analysis of Copy number alteration by Expression (ACE) algorithm was developed to identify amplified or deleted chromosome regions based on gene expression data[13]. While this approach demonstrated the utility of leveraging expression data to identify candidate CNV regions and genes whose expressions might be affected by the candidate CNV, the identified regions were often large and harbored many genes. Furthermore, no objective mechanism was employed to distinguish cancer driver genes from passenger genes within a putative CNV region.

Here we describe a novel Wavelet based Analysis of Copy number alteration by Expression (WACE) algorithm and combine it with Bayesian networks via a key driver analysis to provide a systematic and unified approach for distinguishing cancer driver genes from passenger genes residing in inferred copy number variation sites.

## Methods

### Preprocessing data

We collected four independent breast cancer datasets, NKI[14], Wang[6], Miller[15], and Christos[8]. NKI samples were profiled on the Agilent Human 25 K platform comprised of 24,496 non-control oligonucletoide probes while the other studies were carried out using the Affymetrix HG133A platform, compromised of 22,282 probe sets. Each of the four microarray datasets was adjusted for estrogen and progesterone receptor (ER/PR) status as well as age to avoid their influence. The data were fit using a robust linear regression model (rlm function from R statistical package), and the residuals with respect to the model fit were carried forward in all subsequence analyses as the gene expression traits. In all analyses except ACE, the expression of individual gene/probe was used. In the subsequent analysis, for a gene with multiple probes we used the average expression profile to represent its expression so as to eliminate the weight effect due to multiple data points on a single location.

We also downloaded the gene expression and aCGH data from the Stanford University Breast Cancer Study http://smd.stanford.edu/[16]. Again the expression profiles of multiple probes representing the same gene were consolidated by average. The expression and aCGH data were adjusted for array batch, ER/PR status and age to avoid their influence.

### A Unified Framework for Expression based CNV Inference and Causal Gene Identification

**Figure 1:**
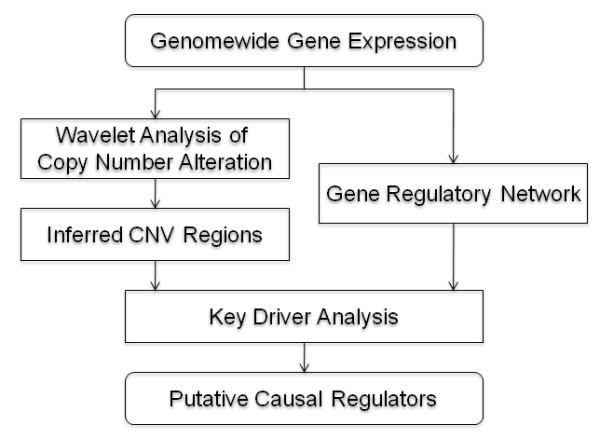
**A framework for integrating wavelet based CNV inference and gene network analysis**. The samples in a given gene expression study are first partitioned into two groups based on phenotypes such as poor versus good outcome, followed by differential expression analysis (t-test) to yields expression scores (ES t-statistics). Wavelet analysis is then performed on ES' ordered by gene chromosomal locations to detect significant consecutive regions (called inferred CNV regions). Using the same gene expression data, a gene regulatory network (Bayesian network) is constructed. Finally, the inferred CNV regions and the Bayesian network are input to the key driver analysis to identify potential cancer driver genes.

We developed a framework for integrating CNV inference and gene regulatory network analysis to provide a systematic and unified approach to prioritizing genes residing in inferred CNV (ICNV) regions (Figure 1). Under this framework, we first developed a wavelet-based ACE algorithm (WACE) to more efficiently and accurately detect amplified/deleted regions of the genome in cancer samples. For a given gene expression study the samples are first classified into two groups based on phenotypes such as poor versus good outcome and the two corresponding subsets of gene expression data are then taken as input for WACE to infer significantly amplified or deleted CNV regions. Meanwhile, a regulatory network is constructed using the Bayesian network reconstruction method. Finally, genes on the inferred CNV regions are input into a key driver analysis to identify hub genes in the network as potential cancer driver genes. When multiple datasets are available, all ICNV regions are aligned to form recurrent CNV regions and multiple regulatory networks are combined into a single network. The recurrent CNV regions and the combined network then go through the key driver analysis to identify putative regulators.

### Wavelet based Analysis of Copy number alteration by Expression (WACE)

**Figure 2:**
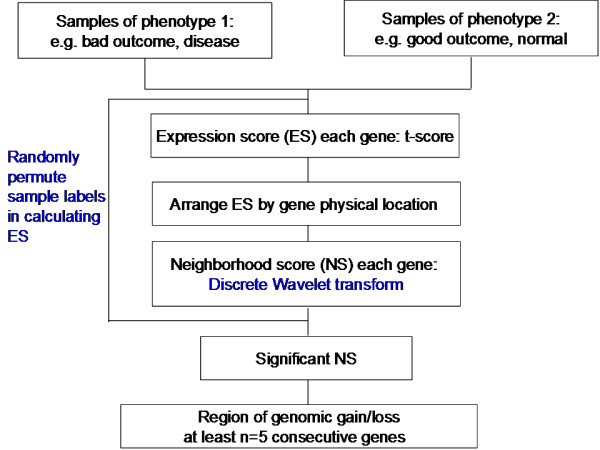
**Outline of the WACE algorithm**. For a given gene expression dataset, the samples are classified into two groups based on phenotypes such as poor versus good outcome and the genes are ordered based on their physical location on chromosomes. Expression scores (ES, t-statistics) for all the genes are computed and then subjected to wavelet transform to obtain smoothed ES, called neighboring score (NS). The significance (false discovery rate, FDR) of NS on each individual chromosome is empirically approximated based on its null distribution by performing the same wavelet transform on "random" ES's based on the randomized samples. A segment containing at least *n *consecutive positive/negative NS with FDR ≤ 0.01 is defined as an inferred CNV region. ICNV regions from multiple datasets are finally aligned to determine the recurrent regions of CNV.

WACE algorithm is described in detail in the Method of Additional File 1 and summarized by a diagram in Figure 2. In brief, for a given dataset, the gene expression traits were first ordered according to their physical location on chromosomes. The expression scores (ES, t-statistics) were computed for each gene with respect to the good and bad tumor outcome, and were then subjected to wavelet transform to obtain the neighboring scores (NS). To evaluate the significance of the NS's on each individual chromosome, we empirically approximated its null distribution by applying wavelet transform to "random" ES's based on randomizing the sample class labels with respect to the expression vectors, repeating this process 1000 times. The false discovery rate for each observed NS was computed as the fraction of random NS's that were greater than (less than) or equal to the observed value if NS > 0 (NS < 0). After evaluating the statistical significance of NS, an ICNV region on a chromosome was identified if it harbored at least *n *consecutive positive/negative NS's at a false discovery rate < 0.01. The n value, which varied from 5 to 10, was proportional to the scaling level used in wavelet transform which in turn relate to the gene/probe density of the microarray platform. A high scaling level of wavelet transform increases the NS magnitude of neighbor points around a single differentiated gene, and thus makes them become statistically significant, which might in turn falsely identify a region as ICNV if n is small (Additional File 1, Figure S1B). Therefore, n value is determined by the scaling level used in wavelet transform. The higher scaling level requires large n. We have tested different n values from 5 to 10 for a given s = 5 in the Stanford data which is comprised of both aCGH and expression data. We have found that n = 10 provides the best overlap between aCGH-and expression-based expression. Similarly, n = 5 which corresponds to s = 3, provides robust results for identifying recurrent ICNV regions. Finally, ICNV regions in multiple datasets were aligned to determine the recurrent CNV regions. The selection of filter function and scaling level for wavelet transform, as well as the validation of the method, is discussed in detail in Additional File 1.

### Distinguishing cancer driver genes from passenger genes via reconstruction of Bayesian networks

**Figure 3:**
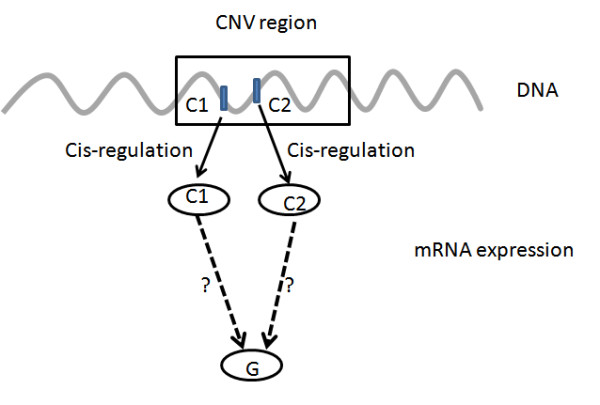
**Cancer driver genes versus passenger genes**. Two candidate genes C1 and C2 are located on an inferred CNV region, and are likely cis regulated by a CNV. To distinguish potential drivers from passengers, we test whether they can causally regulate other genes. To determine whether a downstream gene G is regulated by C1 or/and C2 or neither is equivalent to selection among four competing models. Given that many other potential genes or factors affect the expression level of gene G, the models conditioning on all other factors are needed to evaluate, i.e., a Bayesian network reconstruction process.

CNV regions harbor many genes but only a small portion of them are cancer causal genes. A key challenge in cancer genomics is to distinguish the cancer driver genes, which are causal for oncogenesis and whose variations confer growth advantage on cancer cells, from the passenger genes that are physically located close to the driver genes in CNV regions[17]. Several methods have been proposed to indirectly identify driver genes by intersecting genes associated with CNV regions and gene coexpression networks[18] or protein-protein interaction data[19]. In our study, gene expression of two neighbor genes in an inferred CNV region (shown in Figure 3) are correlated to the inferred CNV (as implicated by the WACE method). To distinguish the potential cancer drivers, we tested whether they can causally regulate other genes instead. As shown in Figure 3, two candidate genes C_1 _and C_2 _are cis regulated by a common CNV. To determine whether a downstream gene G is regulated by C_1 _or/and C_2 _or neither is equivalent to select among competing models:(1)(2)(3)(4)

where eq. (1) represents G independent of C_1 _and C_2_, eq.s (2) - (4) represent G regulated by C_1 _, C_2_, both C_1 _and C_2_, respectively. As there are many other potential genes or factors that can affect the expression levels of gene G, the models eq. (1) - (4) are needed to condition on all other such factors. Thus, the model selection process is equivalent to a Bayesian network reconstruction process, which will be discussed subsequently.

Bayesian network is a probabilistic representation of the gene regulatory network and has shown superior performance in integrating genetic data into gene causal networks predictive of complex phenotypes[20-25]. 6,312, 6,349, 6,268 and 5,802 differentially regulated genes for NKI[14], Wang[6], Miller[15], and Christos[8], respectively, were selected as input into a Bayesian network reconstruction software program based on a previously described algorithm[24]. Markov Chain Monte Carlo (MCMC) simulation was then employed to identify the most plausible structures. For each seed, 15*n^2 ^iterations of MCMC were run on average, where n is the number of nodes. The Bayesian Information Criterion (BIC) scores were used as the optimization criteria. One thousand Bayesian networks were reconstructed using different random seeds to start the reconstruction process. From the resulting set of 1000 networks generated by this process, edges that appeared in greater than 30% of the networks were used to define a consensus network. The cutoff of 30% was based on a simulation study[26]. When summarizing 1000 constructed structures from random seeds, the histogram of the number of link occurrences among 1000 structures shows a bimodal distribution, where 30% is the best value to separate the two modes and also has the best recall-precision tradeoff [26]. Edges in this consensus network were removed if 1) the edge was involved in a loop, and 2) the edge was the most weakly supported of all edges making up the loop.

### Key Driver Analysis (KDA)

One primary goal of gene network analysis is to identify key regulatory components, or key drivers, of sub-networks with respect to varying biological contexts [25,27]. The KDA takes as input a set of genes (**G**) and a gene causal (directed) network **N**. The objective is to identify the key regulators for the gene sets with respect to the given network. Candidate drivers are identified as follows. We first compute the size of the h-layer neighborhood (HLN) for each node. The range of h is from 1 to the diameter of the network **N**. Specifically, for a given node *g*, the size of its HLN is the number of its downstream nodes that are within h edges away from *g*. For the given network **N**, let ***μ ***be an array of the sizes of HLNs and ***d ***be an array of the out-degrees for all the nodes. The nodes are nominated as candidate drivers if their sizes of their HLN are greater than , where  is the mean of ***μ ***is and *σ*(*μ*) is the standard deviation of ***μ***. The candidate drivers without any parent node (i.e., root nodes) are nominated as global drivers while the rest are local regulators. Let  be the mean of ***d ***and *σ*(*d*) be the standard deviation of ***d***. We also promote hub nodes as global drivers, i.e., the nodes with out-degrees above  are designated as global drivers. These criteria identify genes with number of downstream nodes or number of outlinks significantly above the average.

By definition, each driver modulates a set of genes, i.e., its downstream nodes. Previous work has shown that a gene's function can be predicted by its neighbor genes in networks[28]. Moreover, a series of validation experiments show that the downstream nodes of a driver predicted by Bayesian networks significantly overlap with its knockout signature[25].

### siRNA Screen and Cell Viability Assays

#### Cell lines and siRNA library

Four different breast cancer cell lines, ER-positive MCF7 and ZR-75-1; and ER-negative MDA-MB-231and MDA-MB-468[29], were obtained from the American Type Culture Collection, Rockville, MD (Catalogue numbers HTB-22, HTB-26, HTB-132 and CRL-1500, respectively). The siRNA library targets ~2,400 unique human genes, with three siRNAs per gene, as described previously[30]. The library represents genes comprising kinases, membrane proteins, enzymes, components of major cellular pathways including cell cycle, transcription regulation, and signal transduction, etc. [31]. siRNA sequences were designed with an algorithm developed to increase efficiency of the siRNAs for silencing while minimizing their off-target effects [32]. siRNAs were ordered from Sigma-Proligo (The Woodlands, TX).

#### siRNA screen and cell viability assays

siRNA screens were performed as described previously[30] and cells were transfected using RNAiMAX (Invitrogen, Carlsbad, CA). Cell viability assay was determined using AlamarBlue reagent (BioSource International, Camarillo, CA). The fluorescence signal was corrected for background (no cells). Cell growth was expressed as % viability relative to the median value of wells transfected with an siRNA to Luciferase.

## Results

As detailed in the Materials and Methods, the proposed unified framework for expression based CNV inference and causal gene identification is comprised of three major components, wavelet based CNV inference, causal network construction and key driver identification.

### Performance Comparison of WACE and GACE

The original ACE approach for identifying amplified or deleted chromosome regions used the simple Gaussian transform to smooth the data and then identified the significantly abnormal regions comprised of over- or under-expressed genes via a permutation test[13]. Although this approach, named GACE, was able to narrow down genes whose expression might be affected by the local CNV, it often systematically overestimated the size of the identified regions which were typically re-arrangements of small sequences. We improved GACE by introducing: i) a wavelet based smoothing technique and ii) a new statistical method for assessing significance of putative CNV regions. Wavelet transform is a more sophisticated filtering technique and has become a cutting-edge technology in signal and image processing because of its superior ability to accurately deconstruct and reconstruct finite, non-periodic and/or non-stationary signals[33]. This led to a wavelet-based ACE algorithm (WACE) to efficiently and accurately narrow down amplified/deleted regions of the genome in cancer samples.

To access the performance of WACE, first we compared it with the existing method GACE based on a previously published breast cancer study [16], which consisted of gene expression and aCGH data, as well as clinical data relating to tumor progression (referred to here as BCS1). A detailed comparison is described in Additional File 1, WACE and GACE Comparison (Section 2.1). Figure S4 to S6 in Additional File 1 illustrated the comparison results between two methods based on (i) the correlation coefficient between the expression- and aCGH-based NS profiles, and (ii) re-identifying CNVs based on gene expression, respectively. Here we summarize the findings: (i) WACE uncovered almost three times as many expression ICNV regions overlapping with the aCGH ICNV regions compared to GACE, and (ii) these two sets of regions identified by WACE were better correlated with each other than those identified by GACE.

We also verified the cis-effect of CNV on gene expression by considering the correlation between the aCGH and gene expression data of each gene in BCS1. The expression of the genes located in the overlapped CNV regions was highly correlated to the corresponding aCGH data. For instance, WACE identified in both expression and aCGH data a DNA amplification region at 20q13, and the gene expression and aCGH NS profiles were highly correlated (r = 0.55).

### Amplified regions associated with poor outcome affect cell cycle

We applied WACE to the aforementioned four breast cancer microarray studies [6,8,14,15] to infer CNV regions associated with metastasis. Each dataset was independently analyzed by WACE and the identified regions were then aligned to locate the recurrent regions of ICNVs. To calculate the ES's for each dataset, the samples were classified into two groups, the patients with metastases within 5 years and those with no metastasis after more than 5 years of follow-up.

**Figure 4:**
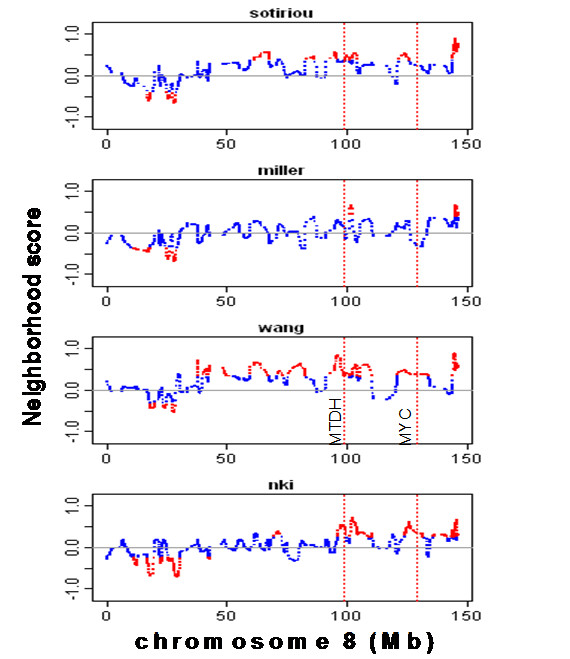
**NS profiles on chromosome 8 from the four independent breast cancer studies using WACE**. Red lines encode identified CNV regions. *MYC *neighborhood region (121-133 Mb) is detected to be amplified in two studies by WACE but only in one study by GACE.

Figure 4 shows the NS profiles on chromosome 8. WACE identified the amplified chromosome 8q21-q23 region (around 100 Mb and harboring the *MTDH *gene) in three of the four datasets. The amplification of this region has previously been experimentally verified [13]. It also identified CNV in the 8p21-p12 and 8q24.3 cytobands associated with tumor outcome. The 8q24 cytoband, which was consistently detected as an amplified region by WACE, was found to be the most frequently amplified region by genome-wide array CGH[9,12], where the expression of the underlying genes also reflected the change in DNA copy number[12]. Moreover, this region also includes the well-known oncogene *MYC*.

**Figure 5:**
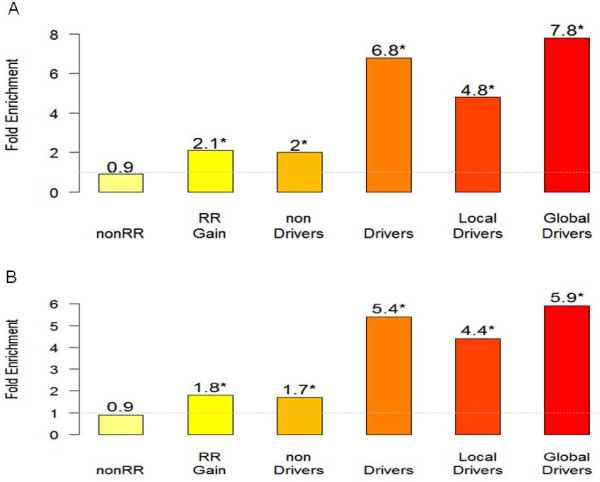
**Validation of predicted key drivers by testing the enrichment of siRNA hit signatures in various gene sets**. Two siRNA hit signatures, V1 (an across cell line signature with 216 genes) and V2 (with 484 genes as a union of the signatures across cell lines and from the individual cell lines), were tested for enrichment in the following gene sets: the genes not on the recurrent regions (nonRR), the genes on the amplified ICNV regions (RR Gain), the non-driver genes (non Drivers) on the amplified regions, the drivers (global and local), the local drivers and the global drivers. The drivers and non-drivers were based on the key driver analysis on the amplified regions and the Bayesian network. A) and B) shows the fold enrichment of V1 and V2 signatures in the gene sets, respectively. The genes on the recurrent ICNV regions are more than twice more likely to enriched for the siRNA signatures than the genes not on the recurrent regions while the global drivers has the highest likelihood (7.8 and 5.9), followed by the drivers (6.8 and 5.4), the local drivers (4.8 and 4.4), and the non drivers (2 and 1.7).

The recurrent regions of CNV were defined as the union of the regions in which the abnormal gain/loss events were identified in at least two out of the four studies. WACE found 109 recurrent regions covering 2,560 genes (Table 1 and Additional File 1, Table S1). Table 1 lists the recurrent regions of amplification that were consistently found by WACE in all four datasets. Several regions on chromosome 8, 11, and 20 identified here have been previously identified via analysis of CGH arrays applied to breast cancer tumors [10]. These results further confirm the accuracy of WACE. We note that many of the genes in the inferred recurrent regions were oncogenes (19 of total 84 oncogenes according to Panther database) or significantly affected the cell viability based on siRNA experiments (Figure 5).

**Table 1:** The inferred recurrent regions of CNV in the four breast cancer datasets.

Cytoband Location	Chr	Start(Mb)	End (Mb)	Size (Mb)	Gain/Loss	Important genes
chr3q26.31-q27.1	3	173.95	185.91	11.96	G	ECT2, PIK3CA, PSMD2, CLCN2, POLR2H

chr4p16.3	4	0.69	1.95	1.26	G	CTBP1, TACC3

chr5q35.2-q35.4	5	173.41	179.09	5.69	G	GRK6, DDX41

chr8p21.2-p12	8	23.59	30.70	7.11	L	CLU

chr8q24.3	8	143.54	146.25	2.71	G	NRBP2

chr11q13.1-q13.4	11	64.54	71.59	7.05	G	MAP3K11, ADRBK1, RPS6KB2, LRP5, CPT1A

chr12q24.23-q24.33	12	120.70	122.32	1.62	G	

chr16p13.4	16	2.02	2.66	0.64	G	DCI, PDPK1

chr16q12.2-q22.1	16	54.25	69.11	14.87	G	POLR2C, KIFC3, CSNK2A2, GOT2, CDH5, PSKH1, PSMB10, VPS4A,

chr16q22.3-q24.3	16	82.56	88.62	6.06	G	GALNS

chr20q11.1-q11.23	20	28.24	36.99	8.75	G	TPX2, APBA2BP, E2F1, EIF2S2, AHCY, GSS, PROCR, RBM39, SCAND1, DLGAP4, GHRH, BLCAP

chr20q13.12-q13.13	20	44.43	49.01	4.58	G	ARFGEF2, CSE1L

The important genes include known oncogenes, known tumor suppressors, and the genes that, when silenced, reduced cell viability by more than 50% based on our siRNA experiments.

These regions were tested for gene enrichment using the gene ontology (GO) categories, KEGG, Panther, and GeneGo pathways. The most enriched categories include nucleosome assembly (P < 7.24E-22), chromatin assembly (P < 1.02E-20), systemic lupus erythematosus (P < 9.03E-18), chromatin assembly or disassembly (P < 4.57E-17), DNA packaging (P < 1.29E-16) and protein-DNA complex assembly (P < 1.92E-11). Cell cycle and cholesterol biosynthetic process pathways are also enriched in the regions. All the enriched categories are shown Additional File 1, Table S3.

### ICNV regions versus aCGH based regions

We further examined to what extent the recurrent CNVs inferred by gene expression reflect the recurrent CNVs based on an independent aCGH data in BCS1. To ensure an objective comparison, we used a popular CNV detection method, the circular binary segmentation (CBS) algorithm (implemented in the DNAcopy package from Bioconductor) on the aCGH data. Forty six or 68.7% of the 67 CBS based aCGH CNV regions in BCS1 significantly (gene-based FET P = 3.1e-13) overlapped with the 109 recurrent ICNV regions detected by WACE on the four gene expression studies. The overlapped regions on chromosome 8, 11, and 20 have been previously identified via analysis of CGH arrays applied to breast cancer tumors [10]. These results demonstrate a good correspondence between the inferred CNV regions and the experimental results and thus confirm the accuracy of WACE.

### Breast Cancer Gene Regulatory Networks

Four whole-genome gene regulatory networks were first constructed by the Bayesian network reconstruction method [26] for the aforementioned four breast cancer studies (NKI, Christos, Miller, and Wang) and then were combined by union of directed links to form a single network, which consisted of 10,118 genes and 20,732 directed links. Previous works have shown that genes controlling many other genes in gene regulatory networks are more likely regulatory genes regulating their downstream genes[24,25,27]. The top 5 genes with the most out links are *ARF1, FOXA1, ESR1, HIF1A *and *UBE2C*. *ARF1 *is involved in the activation of the PI3K/AKT pathways which regulate cell survival and proliferation[34]. *HIF1A *plays an essential role in cellular and systemic homeostatic response to hypoxia was recently found to regulate the metastasis of breast tumor to lung[35]. *FOXA1*, *ESR1 *and *UBE2C*, are known as breast cancer prognosis biomarkers[36]. The global features of the combined network are described in detail in Additional File 1, Properties of Breast Cancer Bayesian Networks (Section 3).

### Key Driver Analysis

We assume that the cancer driver genes have broad impacts on global gene expression while passenger genes have small impacts [37]. To quantify impacts of genes residing in the recurrent ICNVs detected across multiple data sets, we count numbers of downstream genes that are regulated by each candidate gene locally or globally in the combined network. Genes are grouped into global drivers, local drivers and passengers according to the rank of these numbers (see Methods for details). In the key driver analysis, we focus on the genes residing in amplified regions which are regarded as a hallmark of dominant cancer driver genes[17].

For the genes in the recurrent regions of amplification (RR Gain), we identified 44 global drivers and 24 local drivers. Known breast cancer susceptibility genes like *TPX2*, *AURKA*, *TK1 *and *BIRC5 *are among the top 7 global drivers (the other 3 top drivers are *GINS2*, *COX4NB *and *NUP93*). Other known breast cancer gene target such as *ERBB2*, *E2F1 *and *MTDH *are in the global driver list. Many of them such as aurora kinases *AURKA *and survivin (*BIRC5*) have been targets for anticancer drugs [38-40].

To access the robustness of predicted key drivers, we performed the KDA on the networks (named as the 3N networks) as combinations of any three of the four Bayesian networks. Over 79% of the drivers based on the 3N networks are the drivers based on the combination of all the four BNs. On the other hand, the four driver sets based on the 3N networks significantly overlap with each other (with FET p-values < 1E-14) and they share at least 41% of their members. Therefore, the drivers uncovered are fairly robust. It is of note that the network based on all the four networks leads to many more drivers than any 3N network, indicating the great advantage of the combination of all the networks.

### Validation of key drivers via in vitro siRNA knockdown experiments

We assessed the effect of knocking down the genes located on the amplified recurrent ICNV regions by siRNA on breast cancer cell viability because they were enriched for gene sets involved in cell cycle and metabolic regulation. In the previously described siRNA experiments, the median of the viability of the siRNA transfected cells was 64.30%, with a standard deviation of 15.28%, compared to the control cells transfected with an siRNA to luciferase. We used a viability cutoff value of 41.38% (1.5× standard deviation below the median) to determine viability signature genes that, when silenced, significantly decrease cell viability. This resulted in the identification of a 216 gene signature (V1) with a significant effect on cell viability in multiple cell lines. Based on a cutoff of 1.5× standard deviation below the median, we also derived a signature for each cell line. The four signatures from individual cell lines were then combined with V1 to derive a combined signature V2 with 484 genes. The genes in V1 and V2 and their viability scores are shown in Additional File 2, Table S4.

We assessed whether V1 and V2 signatures are significantly enriched in different gene sets including the global drivers, the local drivers, the drivers (global and local), the non-driver genes and the genes not on the recurrent regions (nonRR). Figure 5 shows the fold enrichment of each gene set for the siRNA hit signatures V1 and V2. When compared with the genes not on the recurrent ICNV regions, the genes on these regions are about twice (i.e. 2.1 and 1.8 vs. 0.9) more likely to fall into V1 and V2, respectively. Also the global drivers are most likely to be siRNA hits in both V1 and V2, followed by local drivers and non-drivers. For example, the global drivers, the local drivers and the non drivers are 7.8 (P < 5.8e-4), 4.8 (P < 1.9e-2) and 1.97 (P < 2.5e-6) times more likely to overlap with V1, respectively than the genes on the human genome. When the genes on the recurrent ICNV regions were considered as background, the global drivers were 4.2 (P < 0.0055) and 3.5 (P < 0.003) times more likely to be in V1 and V2, respectively, and the local drivers are less significantly enriched for the signatures.

**Figure 6:**
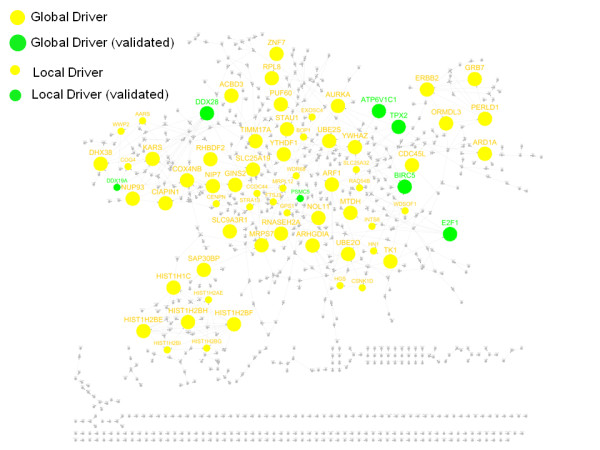
**A regulatory network for the genes on the amplified recurrent ICNV regions**. The key driver analysis was applied to the genes on the regions and the combined Bayesian network to identify potential key drivers. The largest nodes in the network are the global drivers and the medium size nodes are local drivers while the non-drivers are the smallest nodes. The genes found in the siRNA signature V1 are highlighted in green.

Seven drivers (*ATP6V1C1, BIRC5, DDX19A, DDX28, E2F1, PSMC5 *and *TPX2*) are in V1. The protein Survivin is encoded by *BIRC5 *(located in 17q25) and is well-known for its role in mitotic regulation and apoptosis, and has been developed as a target for cancer treatment [39]. Silencing *BIRC5 *decreased the cellular viability by 64%. *TPX2 *is located on 20q11 and the cell viability dropped by 64% when it was silenced. Figure 6 shows the subnetwork associated with the amplified recurrent ICNV regions. The global and local drivers are highlighted in different sizes. The genes found in the signature V1 are highlighted in green.

As mentioned earlier, *AURKA *and *TPX2 *are the top key regulators of the recurrent ICNV regions. On the other hand, 107 of the genes on the recurrent regions overlapped with V2 and they include *TACC3*, *ECT2, CDC6, NOTCH1*, and *PIK3CA *etc. Here we found a molecular mechanism that linked *TACC3 *and *TPX2 *through *AURKA*. *TACC3*, located on an amplified region on 4p16, recently emerged as an important gene in the stabilization of the mitotic spindle[41], and is up-regulated in several cancer cell lines including thyroid and lung cancers[42-44]. Cell viability was reduced by 59.80% in the *TACC3 *siRNA transfected cells. *AURKA *resides on 20q13, which was also identified as a recurrent region of amplification. It has previously been shown that *TPX2 *is one of several activators for *AURKA *[45], which in turn controls the localization of *TACC3 *to the spindles [46]. Furthermore, *TPX2 *acts as an allosteric activator of Aurora A, and together they inhibit the activity of tumor suppressor p53 in *Xenopus Oocytes *(Pascreau et al. 2009). The molecular interaction between the hubs located on different recurrent regions might suggest the trans-association between regions. We expect more mechanisms of this kind will be unraveled through the integration of WACE and network analysis.

These experimental results demonstrate the importance of the recurrent ICNV regions and the predicted key driver genes, especially global drivers in terms of significant impact on cancer cell viability, and thus validate our procedures for inferring CNV regions and identifying cancer driver genes in these inferred CNV regions.

## Discussion

We developed a novel Wavelet based algorithm to Analyze Copy number alteration based on Expression (WACE) and further combined this analysis with gene regulatory network analysis to identify the cancer driver genes in the chromosome regions with genomic alterations. The performance of these two methods was evaluated in Additional File 1, Section 2. In brief, WACE improved on the state-of-the-art algorithm (GACE) by (i) reducing the over-smoothing effect of the Gaussian transform and retaining the local patterns, and (ii) eliminating the type I and II errors induced by the non-zero centered null distribution (Additional File 1, Figure S2). WACE demonstrated superior performance over GACE on several large scale gene expression datasets (see Additional File 1, Section 2). By analyzing multiple datasets, we were able to identify recurrent ICNVs that harbor many cancer-related genes, suggesting genes in these regions are worthy of further study.

Integration of the recurrent regions identified by WACE and the gene regulatory network analysis uncovered not only many well-known oncogenes like *BIRC5*and *E2F1*, but also a number of novel cancer susceptibility genes such as *ECT2*, *TPX2 *and *TACC3*, which are involved in cell cycle and ECM regulation. Many of the cancer driver genes we predicted were subsequently validated via siRNA experiments. Table S5 in Additional File 1 includes the four known and 45 novel breast cancer genes that are in the recurrent regions of amplification and validated by the siRNA experiments across multiple breast cancer cell lines.

The importance of the identified recurrent regions of amplification (ARR) and the inferred drivers are further justified by another independent dataset from Cancer Gene Census http://www.sanger.ac.uk/genetics/CGP/Census/. Among the 19 known breast cancer genes compiled by Cancer Gene Census, 5 are located in the ARRs while 14 are outside the ARRs, indicating that the known breast cancer driver genes are 3.5-fold overrepresented in the ARRs when compared to the genes not on these regions. Among the 5 census genes located in the ARRs, one (*ERBB2*) is the global key driver and the rest four are not the drivers, representing 25- and 2-fold enrichments, respectively, compared to randomly selected human genes.

There are multiple molecular factors contributing to poor outcome, including cell proliferation, metastasis and cell stemness etc. Our assumption is that CNV significantly contributes to the disease outcome though the separation of the samples into good and bad outcome groups tends to identify metastasis genes. We don't need to pre-specify through what molecular mechanism that CNV drives disease outcome, which can be inferred in a data-driven manner. To further understand the functions of the ARRs, we performed gene co-expression network analysis[47,48] on the four breast cancer datasets to identify gene modules (comprised of highly interacted genes). A module enriched for cell cycle genes (named as cell cycle module) and conserved across the four corresponding networks is most predictive of survival in all the four studies, indicating cell proliferation contributes to breast cancer outcome. The ARRs are highly enriched for the genes of the cell cycle module (P < 1.2e-14). This is further supported by the fact that the inferred key drivers include not only known breast cancer metastasis genes (*MTDH*[13] and *ERBB2*[49]) but also known cancer cell cycle genes such as *TPX2*, *AURKA, E2F1 *and *BIRC5*. As shown in Figure 6, these known cell cycle and metastasis genes interact with each other and with many other drivers to co-regulate the genes on the AARs. Furthermore, AARs are enriched (2.3 fold-enrichment, P < 2.6e-2) for a cell cycle prognostic signature identified as metastasis markers[50], and two (*AURKA *and *BIRC5*) of the thirty-three signature genes are the global key drivers (32 fold-enrichment, P < 1.8e-3). Therefore, cell viability assay, used to test cell proliferation, at least partially validated our drivers. The future experiments will consider cell mobility assay to test how these inferred key drivers impact cancer cell mobility that drives metastasis.

A recent paper by Akavia et al. describes a method to predict cancer driver genes by integrating CNA and gene expression data[51]. Akavia et al's approach bears certain similarity as ours. Both approaches first define gene groups of interest, then identify genes in the groups with cis-CNV as candidate driver genes. As we discussed in the main text, expression levels of genes in the same CNV segments are likely to be correlated so that they are assigned into the group. Candidate genes (with cis-CNV) in the same co-expression module are equivalent so that we cannot rank which candidate genes are more likely to be drivers. Akavia et al. used a literature based method to rank candidate genes. Basically, they chose genes with known connections to cancers. However, our approach is completely data driven. We first constructed causal networks to dissect how genes in the regions of interest are regulated and related to each other and then we ranked candidate genes based on the causal networks, which is the key driver analysis described in the main text. As we mentioned in the previous discussion, majority of the drivers identified by our approach are novel and thus they won't be picked up by Akavia et al's literature based approach.

Jornsten et al. recently developed the EPoC method to integrate CNA and gene expression variations[52]. EPoC focuses on CNA driving gene expression changes but disregards the indirect changes through more hubs. Only genes with CNA can be cancer driver genes based on the EPoC model. However, our Bayesian network approach focuses on how genes are causally related to each other. In our case, predicted cancer driver genes may or may not have copy number alterations. Theoretically, the EPoC method can also derive gene-gene relationship as Jorsten et al. indicated that gene-gene relationship matrix ***A ***is the inversion of the CNA-gene relationship matrix ***G ***(***G ***= ***A***^-1^). However, they showed that there was no prediction power using the **A **matrix. One reason is that in their *Lesso *formulation for ***G ***matrix, they explicitly assumed CNAs are hubs so that ***G***^-1 ^is not exactly ***A***. We have showed that gene-gene networks based on our Bayesian network approach have prediction power[24-27]. Thus, our method can be complimentary to the EPoC method.

The impact of CNV on gene expression has been extensively studied[53,54]. In our analysis, we focused on genes with cis-regulated CNVs. CNVs may result partial deletions of genes and the functional changes of the affected genes in turn cause expression changes of downstream genes. Gene regulatory networks may be able to capture the effect of these cis-regulated CNVs on the trans-regulated ones. In our model, we explicitly assume CNVs cause gene expression changes. There are many mechanisms that CNV can be arisen[55]. It is possible that expression changes of genes in DNA repair processes affect CNV. More sophisticated causal models, such as dynamic Bayesian network, are needed to capture these causal relationships. It is of note that there is a limitation of this transcriptome-based approach. We may miss kinases or enzymes that drive cancer progression and metastasis if these kinases' or enzymes' activity changes are mainly due to protein level changes. Complementary proteomic approaches are needed to complement this approach.

## Conclusion

The framework where the wavelet analysis of copy number alteration based on expression coupled with the gene regulatory network analysis, provides a blueprint for leveraging genomic data to identify key regulatory components and gene targets. To our knowledge, this is the first effort to systematically identify and validate drivers for expression based CNV regions in breast cancer. This integrative approach can be applied to many other large-scale gene expression studies and other novel types of cancer data such as next-generation sequencing based expression (RNA-Seq) as well as CNV data.

## Competing interests

The authors declare that they have no competing interests.

## Authors' contributions

BZ, JZ and ES conceived and designed the experiments. LT and BZ designed the data analysis scheme. CZ, TX, JL, HD and JZ collected samples and preprocessed the data. LT, BZ, ZZ, CZ, TX, JL and JZ analyzed the data. ZZ performed validation experiments. LT, BZ, ZZ, JZ and ES wrote the paper. BZ coordinated the study. All the authors read and approved the final manuscript.

## Supplementary Material

Additional file 1 **Supplementary Information**. Supplementary methods and results. Click here for file

Additional file 2 **Supplementary Table S4**. siRNA screen signatures and the associated viability scores. Click here for file
