# Supplementary material for: Inferring causal genomic alterations in breast cancer using gene expression data
Source: BMC Syst Biol. 2011 Aug 1;5:121. doi: 10.1186/1752-0509-5-121 (PMC3162519; doi:10.1186/1752-0509-5-121)
Supplement: Additional file 1 — Supplementary Information. Supplementary methods and results. [file 1752-0509-5-121-S1.DOC]

**Supplementary Information (SI)**

1. **Method**
   1. **Wavelet Transform**

The wavelet transform is a sophisticated filtering or smoothing technique and has become a cutting-edge technology in signal and image processing because of its superior ability to accurately deconstruct and reconstruct finite, non-periodic and/or non-stationary signals. Different from traditional filtering techniques (e.g. Fourier transform) which are defined on the time space, wavelet transform is defined on the time-scale space [^1]^, as shown below:


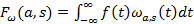
 , (1)

where


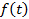
 is a given input signal,


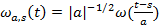
 (2)


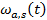
 is a wavelet function at scale *a* and position *s*.

The signal
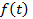
can then be reconstructed again from inverse wavelet transform:


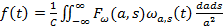
 , (3)

where *C* is a constant.

A scaling level determines the level of decomposition to represent signals at certain resolution. The higher a decomposition level, the lower the resolution of the represented signal. The operation on a time-scale space, a defining characteristics of wavelet functions, provides high localization in both the time and frequency domains and thus allows effective signal reconstruction and representation at multiple resolutions [^1]^.

- 1. **Wavelet Analysis of Copy number alteration by Expression (WACE)**

Given the complex structural patterns in cancer genome and the noisy nature of the high through microarray, wavelet transform fits well the task of uncovering genome-wide true signals or substructures. Recently wavelet transforms have been used to remove noise from microarray data [2-^3]^ and to explore the variation in gene expression clustering [^4]^.

The wavelet-based ACE algorithm (WACE) increases specificity in detecting amplified/deleted regions of the genome in cancer samples as being compared to the original Gaussian transform based method (GACE).

A diagram of the WACE algorithm is depicted in Figure 2. For a given dataset, the gene expression traits were first ordered according to their physical location on chromosomes. The samples were then classified into two groups based on a given clinical phenotype of interest, such as poor versus good outcome, and then the ES’s were computed for each gene with respect to the two groups. The ES’s were then subjected to a smoothing procedure in which neighborhood data points are incorporated in de-noising the point of interest. In our algorithm, we used a wavelet transform to obtain the NS’s. The parameter selection for wavelet transform depends on the density of data, the scale of CNVs region (focal vs. amplicon). The next section would discuss in detail about criteria for selecting parameters in our algorithm. To evaluate the significance of NS on each individual chromosome, we empirically approximated its null distribution by performing wavelet transform on "random" ES's based on the randomized samples with respect to the expression vectors, repeating this process 1000 times. Since the sample class labels were shuffled for every random t-statistics (i.e. ES), the underlying connections between genes and sample classes in the original data were completely destroyed. The false discovery rate of each observed NS then was computed as the fraction of random NS that were greater than (less than) or equal to the observed value if NS>0 (NS<0). After evaluating the statistical significance of NS, an ICNV region on a chromosome was identified if it harbored at least *n* consecutive positive/negative NS with false discovery rate less than or equal to 0.01. Finally ICNV regions in multiple datasets were aligned to determine if they overlap, and if they did, the recurrent regions of ICNV would be the union of the overlap ICNVs.

**1.3. Parameter Selection for Wavelet Transform**

To determine the parameters involved in wavelet transform, we compared the results from different parameter settings for ICNV regions. In general, the transformed signal depends on the selection of the filter and scaling functions, which model the frequency and location, respectively, of the signals to be detected. Chromosome 8 was selected as an example for studying the parameter selection since many studies [5-^8]^ have shown that breast cancer tumors frequently have CNV in this chromosome, and such variations have been shown to alter mRNA levels of the local genes [^7]^.

First we examined the effect of different filter functions. Daubechies functions are the most commonly used function in wavelet transform and thus were used here. We tested three Daubechies orthogonal sets D6, D10 and D20 whose indices refer to the number of polynomial coefficients encoding the wavelet moment, so that the higher the index, the more complex the wavelet function. Figure S1A illustrates the NS profiles on chromosome 8 using different wavelet functions at the same scaling level (s = 3) on the Wang dataset. Although the curves were smoother when using more complex functions, they showed the same ICNV regions with slight shifts at the boundaries of the detected regions. Therefore, this approach was quite robust with respect to the selection of filter functions.

Second we explored the effect of scaling level on detecting the ICNV regions. A scaling level determines the level of decomposition to represent signals at certain resolution. The higher a decomposition level, the lower the resolution of the represented signal. Furthermore, each scaling level also requires a minimal number of available data, such that s ≤ 1+(N-1/)(exp(j)-1) where N is number of data and j is the Deubechies filter levels used. Chromosome 8 on the Wang dataset, for instance, had 458 genes, which limited the maximal scaling level of the wavelet transform to eight. We smoothed the raw ES scores by using the same filter function D10, but at four different scaling levels: s = 2, 3, 4, and 5. Figure S1B shows the chromosome 8 NS profiles at the first three scaling levels. The higher scaling level yielded a better overall global pattern, but at the cost of an attenuated local resolution. For example, the higher scale like s = 4 resulted in a loss of locality while the lower scaling level s = 3 did not only retain the local sensitivity but also capture regional patterns. Because one of the goals of the analysis is to identify the cis genes whose expression is changed due to CNV, the scaling level must be selected to maintain the specificity of the raw signal, as opposed to completely whitening it out as a result of the smoothing process. Therefore, the scaling level should be selected before the correlation coefficients between the raw and smoothed ES became effectively invariant with respect to changes in the scaling level. We suggest the optimal scale was mathematically one point before the curve reached its maximal curvature at which the over-smoothing has happened. In Figure S2, the correlation coefficients between the raw and smoothed ES of four datasets at different scale levels are plotted. The curves had maximal curvature at s = 4, so the scale s = 3 was selected as the optimal scale for all analyses related to the identification of CNV cis regulated genes.

As the four breast cancer datasets contain more than 13,000 genes, or more than double to those in BSC1 (i.e. 6455 genes) on all but Y chromosome, they promised a better resolution for detecting CNV regions without scarification of using high scaling level. We smoothed the ES's using the D10 wavelet function at a scaling level of 3, which was systematically selected based on the variation of the correlation between raw and smoothed ES at the genome level (Figure S2). By using the same criterion, the scaling level s = 5 was used for BCS1 data. The different scaling level used here highlights again the importance of selecting these parameters carefully before analysis.

**1.4. The permutation of class sample labels provided zero-mean null distribution of neighborhood score (NS)**

To access the significance of NS, which was the smoothed t-statistics ES, GACE [^9]^ suggested that the null distribution of NS should be built by shuffling the t-statistics (or expression score, ES). We argued that a null distribution was better constructed based on randomized t-statistics, by randomly assigning class labels to the expression values of each gene. Therefore, to evaluate the significance of the NS’s on each individual chromosome, we empirically approximated its null distribution by applying the wavelet transform to "random" ES's based on randomizing the sample class labels with respect to the expression vectors, repeating this process 1000 times. We examined the performance of GACE and WACE on Chromosome 8 based on Wang data, in which the numbers of samples in the two stratified groups were in the same scale (95 and 168 samples for the poor and good outcome groups). The symmetric null distribution generated from the random t-statistics was centered on zero (Figure S3 top panel) while shuffling the t-statistics led to a significantly large positive mean of NS (Figure S3 bottom panel). The permutation of ES location preserved the ES distribution, which was shifted to the right because many genes in the poor outcome group were over-expressed, and that made a subtle shift of "random" NS (smoothed ES). Such a non-zero mean null distribution increases both type I and type II errors in the statistical evaluation of NS, since for the same magnitude, a negative NS could be assumed to be significant, but the respective positive NS was not. This explains why GACE detected more deleted regions than WACE, as discussed in the results.

**1.5. Criterion for identifying Inferred Copy Number Variation (ICNV) region**

The goal of the analysis was to identify the regions of CNV associate with sample traits from gene expression. Therefore, an ICNV region on a chromosome was identified if it harbored at least *n* consecutive positive/negative NS’s at a false discovery rate < 0.01. Figure S1B showed that the high scaling level of wavelet transform increased the NS magnitude of neighbor points around a single differentiated gene, and made them become statistical significant, which might in turn falsely identify region as ICNV if *n* was small. To eliminate such bias created by the high scaling level, the detected ICNV region must be based on large *n* to ensure more than a single gene in the region being differentiated. We recommended the number of consecutive the number of significant NS ranged from 5 to 10 depending on the scaling level *s* of wavelet transform. In this project, we used n = 5 for s = 3, which was used in the four high gene-density breast cancer datasets, and n = 10 for s = 5, which was used in the BSC1 low gene-density dataset.

1. **WACE and GACE comparison**

**2.1. Evaluation of WACE and GACE performance on identifying ICNV regions in BCS1 data which was composed of both mRNA and aCGH**

We applied both the WACE and GACE algorithms to a previously published breast cancer study [1^0]^, which consisted of gene expression and aCGH data, as well as clinical data relating to tumor progression (referred to here as BCS1). The patients in BCS1 were classified into two groups representing good and poor outcomes, based on relapse free survival. The good outcome group was composed of patients who were free of relapse for more than 5 years after diagnosis, while the poor outcome group included patients who had relapsed within 5 years since first diagnosis. There were 10 and 45 samples for the good and poor outcome groups, respectively. Because of the small sample size of the good outcome group, the expression and aCGH datasets were further filtered by removing genes whose data were missing in more than 20% (= 2) of the samples. This filter yielded a total of 6,455 genes representing all but the Y chromosome. GACE and WACE were applied to both the gene expression and aCGH data of this gene set.

We first assessed whether the gene expression imputed DNA variation between the good and poor outcome groups reflected changes based on the direct DNA measurements. We carried out the wavelet and Gaussian transform on raw ES for both aCGH and expression data using different smoothing parameter values, and then compared the smoothed ES profiles of aCGH and expression data. The wavelet transform was decomposed at seven different scaling levels (s = 3 to 9). In the present context the maximal allowable scaling level is defined by the available number of genes on each chromosome. For the Gaussian transform a range of σ^2^ values was explored: σ^2^ = 25, 50, 100, 200, 300 and 400. Although the correlation between the smoothed gene expression and aCGH NSs generated by WACE highly depends on the scaling levels, at scales above 5, WACE led to consistently higher correlations, even when compared to the maximal correlation achieved by GACE, as shown in Figure S4. As discussed in the Methods, both WACE and GACE tended to over-smooth the raw ES at the higher scale levels (i.e., the processed signal became poorly correlated with the original signal and thus lost the specificity of the original one). Therefore, given the dependence of the scaling parameter on the goals of the analysis, careful consideration must be given in selecting the most appropriate scaling level.

As one primary aim of our analysis is to identify cis-regulated genes whose expression levels are influenced by CNV, the scaling level for the wavelet transform must be selected to retain a certain degree of similarity to the raw data. We suggest the optimal parameter is the value before the correlation between raw (ES) and smoothes (NS) become less independent to the variation of the smoothing parameters. In other words, it is the point before the plot of correlation coefficient against smoothing parameter (Figure S2) achieves its maximal curvature at which the over-smoothing has happened. We therefore chose a scale of 5, given the scale with the maximal curvature occurred at s = 6 for WACE, and σ^2^ = 50 for GACE. As depicted in Figure S4, the smoothed gene expression NS was highly correlated with the smoothed aCGH data, with the correlation coefficient equal to 0.43 and 0.49 for GACE and WACE, respectively.

We next examined whether the CNV regions inferred by gene expression significantly overlapped with CNVs inferred by the aCGH data. Because a gene’s expression level is regulated by many factors in addition to the effects induced by cis and trans DNA copy numbers, we would not expect the regional variation observed from the gene expression data to be fully explained by the CNV. WACE and GACE were applied to both aCGH and mRNA data to determine the CNV between the good and poor outcome groups. As discussed in the previous paragraph, the number (*n*) of consecutive genes giving rise to a statistically significant NS depends on the scaling level (*s*). In this analysis, we used *n* =10 consecutive significant NS (FDR < 0.01) for *s* = 5. Although the smoothed gene expression NS was highly correlated to the smoothed aCGH data for both methods, the two methods identified several different CNV regions (Figure S5), likely due to asymmetry in the GACE distribution estimated by permutation testing (Figure S3). GACE tended to generate a non-zero mean distribution of NS and incorrectly identified a number of regions with small NS values (indicated by arrows in Fig S6C and D). Based on the expression data, WACE correctly predicted 38% (i.e. 12 out of 32) of the aCGH ICNV regions, while GACE identified only 14% (i.e. 3 out of 21). Both methods identified ICNV regions from the expression data that were not identified by aCGH. This could be due to the small sample size of the good outcome group (N=10). Or it could reflect other transcriptional regulation mechanisms such as DNA methylation variation, which cannot be detected by CGH arrays.

- 1. **Evaluation of WACE and GACE performance on identifying ICNV regions in four independent expression datasets**

Given the performance of WACE, we applied it to four breast cancer gene expression datasets to infer CNV regions associated with metastasis. Note that only gene expression data were available for these datasets. Each dataset was independently analyzed by WACE and the identified recurrent regions were then intersected to filter out potential false positive regions. We also compared the performance of WACE with that of GACE. The scaling parameter for each method was selected based on the variation of the correlation between raw and smoothed ES at the genome level (See the previous section). For GACE, we used σ^2^ = 50 for the Gaussian function and computed the NS null distribution by shuffling gene locations. The parameter setting for GACE was consistent with the original study [^9]^. For WACE, we smoothed the expression scores using the D10 wavelet function at a scaling level of 3.

For each dataset, the samples were classified based on tumor outcome. The first group included patients with metastases within 5 years and the second group included patients with no metastasis after more than 5 years of follow-up. An abnormal region was defined as a sequence of at least 5 genes giving rise to a significant NS.

Figure S6 shows the WACE-based and GACE-based NS profiles on chromosome 8 for the four breast cancer studies. In general, the GACE algorithm (Figure S6) produced smoother NS profiles and detected fewer but wider CNV regions compared to WACE (Figure S6A). Both methods identified the amplified chromosome 8q21-q23 region (around 100Mb and harboring the *MTDH* gene) in three of the four datasets. The amplification of this region has previously been experimentally verified[^9]^. Both algorithms also identified CNV in the 8p21-p12 and 8q24.3 cytobands associated with tumor outcome. Though the 8q24 cytoband was consistently detected as an amplified region by WACE (Figure S6A), it was missed by GACE (Figure S6B). This region was found to be the most frequently amplified region by genome-wide array CGH [5, ^7]^, where the expression of the underlying genes also reflected the change in DNA copy number [^7]^. Moreover, this region also includes the well-known oncogene *MYC*.

The recurrent regions of CNV were defined as the union of the regions in which the abnormal gain/loss events were identified in at least two out of the four studies. WACE found 109 recurrent regions covering 3497 genes investigated by both Affymetrix and Agilent platform (Table 1 and S1), while GACE detected 73 regions that covered 3596 genes (Table S2). The pie graphs in Figure S7 highlight the portion of the gene sets that was either commonly detected by both methods, or that was uniquely identified by a specific method. In general GACE found more deleted but fewer gained regions compared to WACE. As discussed in the method of SI, the likely reason for this is the asymmetry in the GACE null distribution that obtains from the permutation approach. That is, because genes in the poor outcome group were more frequently over-expressed than repressed, the permutation of gene location retained the imbalance of positive over negative t-scores, resulting in a shift of the mean of the null distribution of the regional scores (i.e. NS) to positive (> 0).

Since the inferred CNV recurrent regions were, by definition, associated with poor outcome, we hypothesized that the underlying genes would relate to cancer biological processes. Therefore, we annotated the gene sets defined by CNV regions using the gene ontology (GO) categories and KEGG pathways. Based on the background of ~13,100 genes whose expression was measured by the Affymetrix platform in these studies, Table S3 lists the genes in the recurrent regions of amplification detected by both methods were enriched for genes in pathways that included nucleosome assembly (fold change for enrichment (FCE) = 7.4, Fisher Exact Test (FET) p < 1.0e-16) chromatin organization (FCE = 2.7, FET p < 1.0e-12), and biosynthesis of steroids (FET p < 1.0e-2). The 903 genes in the amplified regions uniquely detected by WACE were involved in cellular zinc ion homeostasis (FET p = 1.9e-6), controlling G1 phase of mitotic cell cycle (FET p < 2.3e-3), and estrogen receptor signaling pathway (FET p < 3.5*e*-3). Those regions detected only by GACE were enriched for epidermis morphogenesis genes (FET p < 2.3e-4). On the other hand, the recurrent regions of deletion determined by both methods were enriched for genes in cell surface receptor linked signal transduction (FET p < 5.9e-4). The deleted regions uniquely identified by WACE were related to major histocompatibility complex II (MHCII)-mediated immunity (FET p < 5.4e-12), whereas those detected only by GACE were related to cell communication (P<3.5e-10) and collagen metabolic process (P<3.8e-7).

**2.3. Comparison of CNV regions inferred from gene expression with those from independent aCGH data**

Here we examined to what extent the recurrent CNVs inferred by gene expression reflect the recurrent CNVs based on an independent aCGH data in BCS1. To ensure an objective comparison, we used a popular CNV detection method, the circular binary segmentation (CBS) algorithm (implemented in the DNAcopy package from Bioconductor) on the aCGH data. As the goal is to identify CNVs associated with metastasis, aCGH data of 45 samples in the poor outcome group were adjusted by subtracting the respective means of the 10 samples in the good outcome group before going through the detection of CNV regions. The aCGH based CNV regions in each sample were first identified by CBS, and then the regions significantly overlapped in at least two samples were merged to form recurrent CNV regions. A CNV segment was amplified if the average intensity of the segment was > 0.3 (median + 1.67*MAD of the whole data), and deleted if < -0.3 (median - 1.67*MAD of the whole data). We identified a total of 67 aCGH CNV regions associated with metastasis presented in BCS1. The CBS identified CNV regions were then compared to recurrent regions of ICNVs. A CBS-based aCGH CNV region is identified to be overlapped to a WACE-based recurrent region if the number of overlapped genes is statistically significant (Bonferroni adjusted p-value based on FET <0.05).

1. **Properties of Breast Cancer Bayesian Networks**

Four whole-genome gene regulatory networks were constructed by the Bayesian network reconstruction method [1^1]^ for the four breast cancer studies: NKI, Christos, Miller, and Wang. Then, the four networks were combined by union of directed links to form a single network, which consisted of 10,118 genes and 20,732 directed links. On average, each gene has 4 links. Most genes in the combined network have a small number of links while a small number of genes have a large number of links, suggesting the combined network exhibits the scale-free network property. The scale free index [1^2]^ of the network is 0.93 (1 for a perfect scale free network) and the distribution p(k) of the combined network approximates a power law: $p(k)\sim k^{-2.74}$.

1. **Prospective and in vitro validations of prominent recurrent amplicons**

The recurrent regions include many well-known genes that play an important role in breast cancer, as shown in Tables 1 and S1. The genes in these regions share common biological functions, and so, genomic variation in these regions generally could lead to very significant impacts on cellular functions.

*4.1.Cytoband 6p22 (i.e. from 26.07-28 Mb)*

This region was commonly found to be amplified in all four breast cancer datasets studied. Genes residing in this region encode up to 49 proteins of the histone family, making the chromatin assembly and DNA packing biological process the most enriched pathways (P<1e-16 by FET). Our network analysis revealed that the local genes were enriched in Wang's navy, Miller's wheat, and Christos' blue modules. Genes encoding the H2B histone family were identified as the hubs of these three modules. *HIST1H1C*, encoding member 2 of the H1 histone family, was also a hub in multiple networks and its Cox model p-value was significant in the Wang and NKI studies. A more recent study revealed that the H1.2 protein plays an important role in transmitting apoptosis signals from nucleus to mitochondria [1^3]^.

*4.2.Cytoband 6p21.32 (i.e. from 32.27-33.26 Mb)*

This 1-Mb region was identified by WACE as a deletion region in three datasets using the Affymetrix platform and was close to other amplified regions (Table S1). Previous work has shown there is a DNA fragile site located in the region [1^4]^. Breakage at the fragile site may trigger deletions, translocations or amplifications of CNV of the neighboring areas via a breakage-fusion-bridge cycle. These changes in turn could alter the expression of genes in these regions. Genes residing in 6p21 encode many proteins of the human leukocyte antigen (HLA) family, whose frequent loss of heterozygosity (LOH) has been observed in a variety of tumors [1^5]^. Our network analysis showed that the genes underlying this region were enriched in the immune response super-module (Table 2). One of the hubs of the super-module was *PSMB9* involved in antiapoptosis and activation of I-kappaB inhibitor and silencing *PSMB9* by siRNA reduced cell viability by more than 50%.

*4.3.Cytoband 17q25.1-q25.3 (i.e. from 68.79-74.298Mb)*

This DNA segment was identified by WACE as the recurrent region of amplification and was associated with metastasis. Our finding was consistent with the result of another study using aCGH data [1^6]^. Among the 85 local genes, 7 were related to cell cycle (P<7.7e-3), and two (*BIRC5, TK1*) of these 7 were hubs in the cell cycle super-module. *BIRC5*, encoding survivin, is well-known for the regulation of mitosis and apoptosis. *TK1*, thymidine kinase, is an enzyme involved in the synthesis of precursors for DNA and is related to proliferation. The serum level of *TK1* was found to be elevated in breast cancer patients and thus is used as a biomarker for breast cancer [1^7]^.

*4.4.Cytoband 17p13.3 (i.e. from 1.27-1.91Mb)*

The deletion of this short segment was found to be associated with poor outcome in breast cancer cases. Two genes, *SERPINF1* and *HIC1*, were identified as the hub nodes in the extracellular matrix (ECM) super module by our network analysis. Pigment epithelium-derived factor encoded by *SERPINF1* is known to prevent angiogenesis [1^8]^, while HIC1 functions as both a tumor suppressor and a transcriptional repressor [1^9]^. Methylation or allelic loss in ductal carcinoma of the breast was found to be responsible for underexpression of the genes in this region [2^0]^.

*4.5.Cytoband 20q11.1-q11.23 (i.e. from 28.2-37.0.Mb)*

WACE identified not only the gain of 20q13.3 (57-62Mb), known for high frequency of amplification, but also the amplification of the nearby 20q11.1-q11.23 cytoband. Many genes in this region affect cell viability when knocked down (Table1). Its amplification was recently found to play a role in metastasis in several different cancers [21-2^3]^. The genes in this recurrent region are enriched in the cell cycle super-module (P<6.0e-3, Fisher's exact test). The region harbored two important cell cycle hub genes, *TPX2* and *E2F1*. *E2F1* is a member of the *E2F* family of transcription factors that mediate cell proliferation and p53-dependent/independent apoptosis. Silencing E2F1 reduced the breast cancer cell viability by 56%.

*EIF2S2* is another gene in the region that might play a role in tumor progression. It encodes a subunit of eukaryotic translation initiation factor 2, eIF2, which is a translation initiation factor involved in the initiation of protein synthesis. Silencing *EIF2S2* reduced the cell viability by 37%. Recent study showed that *EIF2S2* is related to the development of testicular cancer [2^4]^. Deletion of *EIF2S2* suppressed testicular germ cell tumor in heterozygous agouti-yellow mice.

**Supplement Figures**

Figure S1. **Profiles of neighborhood scores on chromosome 8 of Wang data**. The profiles were obtained by (A) different filter functions: D6, D10, and D20 but same scaling level (s = 3), or (B) by different scaling levels: s = 2, 3, 4 but same filter function D10. The red points indicated the significant NS (FDR<0.01). The neighborhood score (NS) was strongly dependent on the scaling level. The local resolution decreased as increasing the scale level. The scaling level of 4 tended to over smooth the data, while the scaling level of 3 still reserved the local resolution, and reflect the regional variation.

Figure S2: Correlation between the raw and smoothed ES profiles in four breast cancer datasets as a function of scale level of WT.

Figure S4. Null distributions are generated by either permuting sample class labels (top panels) or gene locations (bottom panels) using the gene expression data on chromosome 8 in Wang's study.

Figure S4. Correlation between expression-based NS and aCGH-based NS using WACE (solid line) and GACE (dashed line). The correlation is proportional to the scaling level (WACE) or s2 (GACE). However, the correlation by GACE becomes saturated earlier and never surpasses WACE for s>5.

Figure S5. Neighborhood scores and inferred CNV regions by applying WACE (A and B) and GACE (C and D) to the aCGH (A and C) and the expression (B and D) data in the Stanford University breast cancer study. Red and green lines encode amplified and deleted copy number regions respectively. Permutation of ES used by GACE leads to some small but statistically significant NS (indicated by the brown arrows). WACE correctly predicts more CNV regions (highlighted in pink boxes) from the expression data, compared to GACE.

1. (B)

Figure S6. WACE (A) and GACE (B) based NS profiles on chromosome 8 from the four independent breast cancer studies using GACE.

Figure S7. Commonality and specificity of the gene set detected by GACE (A) method detected many deleted regions while the WACE (B) found more amplified regions.

Table S1. 109 inferred recurrent regions identified by WACE

| segmented | Cytoband Location | Ch | Start (Mb) | End (Mb) | Size (Mb) | Gain/Loss | # of Data sets | Dataset^(1)^ | Genes having growth phenotype^(2)^ |
| --- | --- | --- | --- | --- | --- | --- | --- | --- | --- |
| RR_C1_S65.49_L | chr1p31.3 | 1 | 65.49 | 67.93 | 2.43 | L | 2 | 1;4 | *PDE4B* |
| RR_C1_S70.65_L | chr1p31.1 | 1 | 70.65 | 78.26 | 7.61 | L | 2 | 2;4 | *TNNI3K* |
| RR_C1_S90.06_L | chr1p22.2-p21.3 | 1 | 90.06 | 95.17 | 5.11 | L | 2 | 2;4 | *CDC7* |
| RR_C1_S153.20_G | chr1q21.3-q22 | 1 | 153.20 | 153.80 | 0.60 | G | 3 | 1;3;4 | *HCN3, FDPS* |
| RR_C1_S200.16_G | chr1q32.1 | 1 | 200.16 | 201.16 | 1.00 | G | 3 | 1;3;4 | *PTPN7, UBE2T* |
| RR_C1_S220.86_G | chr1q41-q42.3 | 1 | 220.86 | 234.42 | 13.56 | G | 3 | 1;3;4 | *CAPN2, LBR, PARP1, GUK1* |
| RR_C2_S3.48_G | chr2p25.3-p25.1 | 2 | 3.48 | 10.11 | 6.63 | G | 3 | 2;3;4 | *TUBA4A, STK25, DTYMK* |
| RR_C2_S217.07_G | chr2q35 | 2 | 217.07 | 220.15 | 3.08 | G | 2 | 2;4 |  |
| RR_C2_S233.61_G | chr2q37.1 | 2 | 233.61 | 234.46 | 0.86 | G | 2 | 2;4 |  |
| RR_C2_S241.47_G | chr2q37.3 | 2 | 241.47 | 242.45 | 0.98 | G | 2 | 2;4 | *STK25, DTYMK* |
| RR_C3_S37.26_L | chr3p22.2 | 3 | 37.26 | 38.27 | 1.01 | L | 2 | 3;4 | *ACAA1, MYD88* |
| RR_C3_S45.41_G | chr3p21.31 | 3 | 45.41 | 48.68 | 3.28 | G | 3 | 1;2;3 | *PTPN23* |
| RR_C3_S173.95_G | chr3q26.31-q27.1 | 3 | 173.95 | 185.91 | 11.96 | G | 4 | 1;2;3;4 | *ECT2, PIK3CA, PSMD2, CLCN2, POLR2H* |
| RR_C4_S0.69_G | chr4p16.3 | 4 | 0.69 | 1.95 | 1.26 | G | 4 | 1;2;3;4 | *CTBP1,TACC3* |
| RR_C5_S0.27_G | chr5p15.33-p15.2 | 5 | 0.27 | 9.60 | 9.33 | G | 3 | 1;2;4 |  |
| RR_C5_S38.51_L | chr5p13.1 | 5 | 38.51 | 41.98 | 3.47 | L | 2 | 3;4 | *OXCT1* |
| RR_C5_S53.79_L | chr5q11.2-q12.1 | 5 | 53.79 | 59.82 | 6.03 | L | 2 | 3;4 |  |
| RR_C5_S173.41_G | chr5q35.2-q35.4 | 5 | 173.41 | 179.09 | 5.69 | G | 4 | 1;2;3;4 | *GRK6, DDX41* |
| RR_C6_S21.70_G | chr6p22.3-p22.1 | 6 | 21.70 | 24.83 | 3.13 | G | 2 | 3;4 |  |
| RR_C6_S26.07_G | chr6p22.3-p22.1 | 6 | 26.07 | 26.50 | 0.43 | G | 3 | 1;3;4 | *31 genes encoding histone genes* |
| RR_C6_S27.21_G | chr6p22.3-p22.1 | 6 | 27.21 | 27.97 | 0.76 | G | 2 | 1;4 | *14 genes involved in MHCII-antigen* |
| RR_C6_S32.27_L | chr6p21.32 | 6 | 32.27 | 33.27 | 1.00 | L | 3 | 1;2;3 | *PSMB9, KIFC1* |
| RR_C6_S33.36_G | chr6p21.31 | 6 | 33.37 | 34.66 | 1.30 | G | 2 | 2;4 |  |
| RR_C6_S43.50_G | chr6p21.1 | 6 | 43.50 | 44.34 | 0.84 | G | 2 | 2;4 |  |
| RR_C6_S108.64_L | chr6q21 | 6 | 108.64 | 111.24 | 2.60 | L | 2 | 1;4 | *SMPD2* |
| RR_C7_S0.061_G | chr7p22.3-p22.1 | 7 | 0.06 | 4.80 | 4.74 | G | 2 | 1;4 |  |
| RR_C7_S5.63_G | chr7p22.1-p21.3 | 7 | 5.63 | 7.62 | 1.99 | G | 2 | 3;4 | *PMS2* |
| RR_C7_S71.05_G | chr7q11.22-q11.23 | 7 | 71.05 | 73.31 | 2.26 | G | 2 | 1;4 |  |
| RR_C7_S74.78_G | chr7q11.22-q11.23 | 7 | 74.78 | 75.58 | 0.80 | G | 3 | 1;2;4 | *HSPB1* |
| RR_C7_S90.73_L | chr7q21.13-q21.3 | 7 | 90.73 | 94.67 | 3.94 | L | 3 | 1;2;4 |  |
| RR_C7_S154.72_G | chr7q36.3 | 7 | 154.72 | 158.65 | 3.93 | G | 2 | 3;4 |  |
| RR_C8_S11.39_L | chr8p23.1-p21.3 | 8 | 11.39 | 20.12 | 8.73 | L | 3 | 1;2;4 | *BLK, CTSB* |
| RR_C8_S23.59_L | chr8p21.2-p12 | 8 | 23.59 | 30.70 | 7.11 | L | 4 | 1;2;3;4 | *CLU* |
| RR_C8_S69.03_G | chr8q13.2-q13.3 | 8 | 69.03 | 73.15 | 4.12 | G | 2 | 3;4 |  |
| RR_C8_S93.04_G | chr8q21.3-q23.2 | 8 | 93.04 | 110.65 | 17.61 | G | 3 | 1;3;4 | *MTDH,ATP6V1C1* |
| RR_C8_S121.14_G | chr8q24.12-q24.22 | 8 | 121.14 | 133.76 | 12.62 | G | 3 | 1;3;4 | *MRPL13, MYC, KCNQ3* |
| RR_C8_S143.54_G | chr8q24.3 | 8 | 143.54 | 146.25 | 2.71 | G | 4 | 1;2;3;4 | *NRBP2* |
| RR_C9_S93.02_L | chr9q22.31 | 9 | 93.02 | 94.57 | 1.55 | L | 3 | 1;2;4 |  |
| RR_C9_S128.42_G | chr9q33.3 | 9 | 128.42 | 129.25 | 0.83 | G | 2 | 1;4 |  |
| RR_C9_S134.76_G | chr9q34.13-q34.2 | 9 | 134.76 | 135.31 | 0.56 | G | 3 | 1;2;4 | *TSC1* |
| RR_C9_S137.21_G | chr9q34.3 | 9 | 137.21 | 139.44 | 2.23 | G | 3 | 2;3;4 | *NOTCH1* |
| RR_C10_S0.17_G | chr10p15.3 | 10 | 0.17 | 1.77 | 1.60 | G | 2 | 3;4 |  |
| RR_C10_S43.20_L | chr10q11.21-q11.22 | 10 | 43.20 | 46.51 | 3.31 | L | 2 | 3;4 |  |
| RR_C10_S98.05_G | chr10q24.1 | 10 | 98.05 | 99.33 | 1.28 | G | 2 | 2;4 |  |
| RR_C10_S111.96_L | chr10q25.2 | 10 | 111.96 | 114.20 | 2.24 | L | 2 | 3;4 |  |
| RR_C10_S131.48_G | chr10q26.3 | 10 | 131.48 | 135.22 | 3.75 | G | 2 | 2;4 | *STK32C* |
| RR_C11_S0.40_G | chr11p15.5 | 11 | 0.40 | 0.72 | 0.32 | G | 2 | 2;4 | *HRAS* |
| RR_C11_S64.54_G | chr11q13.1-q13.4 | 11 | 64.54 | 71.59 | 7.05 | G | 4 | 1;2;3;4 | *MAP3K11, ADRBK1, RPS6KB2, LRP5, CPT1A* |
| RR_C11_S95.73_L | chr11q21-q22.1 | 11 | 95.73 | 100.96 | 5.23 | L | 2 | 2;4 |  |
| RR_C11_S119.71_L | chr11q23.3-q24.1 | 11 | 119.71 | 123.12 | 3.41 | L | 3 | 1;3;4 |  |
| RR_C12_S47.68_G | chr12q13.12 | 12 | 47.68 | 48.03 | 0.36 | G | 2 | 1;4 |  |
| RR_C12_S54.63_G | chr12q13.2-q15 | 12 | 54.63 | 55.37 | 0.73 | G | 3 | 1;2;4 | *ERBB3,MIP* |
| RR_C12_S55.77_G | chr12q13.2-q15 | 12 | 55.77 | 63.80 | 8.03 | G | 3 | 1;3;4 | *TBK1* |
| RR_C12_S64.82_G | chr12q13.2-q15 | 12 | 64.82 | 67.52 | 2.70 | G | 3 | 1;2;3 | *MDM2* |
| RR_C12_S107.56_G | chr12q24.11 | 12 | 107.56 | 109.14 | 1.58 | G | 2 | 3;4 |  |
| RR_C12_S117.07_G | chr12q24.23-q24.33 | 12 | 117.07 | 119.39 | 2.31 | G | 3 | 1;3;4 |  |
| RR_C12_S120.70_G | chr12q24.23-q24.33 | 12 | 120.70 | 122.32 | 1.62 | G | 4 | 1;2;3;4 |  |
| RR_C12_S122.72_G | chr12q24.23-q24.33 | 12 | 122.72 | 132.29 | 9.57 | G | 3 | 1;3;4 | *PIWIL1,ULK1,DDX51,POLE* |
| RR_C13_S30.09_L | chr13q12.3-q13.2 | 13 | 30.09 | 33.44 | 3.35 | L | 3 | 1;2;4 |  |
| RR_C13_S35.24_L | chr13q13.3-q14.11 | 13 | 35.24 | 41.43 | 6.19 | L | 3 | 1;2;4 | *DCLK1* |
| RR_C13_S44.87_L | chr13q14.12-q14.2 | 13 | 44.87 | 48.77 | 3.90 | L | 3 | 1;2;4 |  |
| RR_C13_S73.16_L | chr13q22.1-q22.3 | 13 | 73.16 | 76.80 | 3.64 | L | 2 | 1;4 |  |
| RR_C14_S23.61_G | chr14q12-q13.2 | 14 | 23.61 | 23.73 | 0.12 | G | 2 | 2;4 |  |
| RR_C14_S30.43_G | chr14q12-q13.2 | 14 | 30.43 | 34.57 | 4.14 | G | 2 | 3;4 |  |
| RR_C14_S72.46_L | chr14q24.2-q31.3 | 14 | 72.46 | 75.74 | 3.28 | L | 2 | 1;4 | *FOS* |
| RR_C14_S76.81_L | chr14q24.2-q31.3 | 14 | 76.81 | 87.97 | 11.16 | L | 3 | 1;3;4 |  |
| RR_C15_S28.98_G | chr15q13.2-q14 | 15 | 28.98 | 32.18 | 3.20 | G | 2 | 3;4 |  |
| RR_C15_S38.00_G | chr15q15.1 | 15 | 38.00 | 39.36 | 1.36 | G | 3 | 2;3;4 | *RAD51* |
| RR_C15_S57.72_L | chr15q22.2 | 15 | 57.72 | 59.31 | 1.59 | L | 2 | 2;4 |  |
| RR_C15_S72.32_G | chr15q24.1-q24.2 | 15 | 72.32 | 73.10 | 0.79 | G | 2 | 2;4 |  |
| RR_C15_S86.80_G | chr15q25.3-q26.1 | 15 | 86.80 | 87.68 | 0.88 | G | 2 | 2;4 |  |
| RR_C16_S0.17_G | chr16p13.3 | 16 | 0.17 | 0.67 | 0.50 | G | 3 | 1;3;4 |  |
| RR_C16_S2.02_G | chr16p13.4 | 16 | 2.02 | 2.66 | 0.64 | G | 4 | 1;2;3;4 | *DCI, PDPK1* |
| RR_C16_S18.70_L | chr16p12.3 | 16 | 18.70 | 20.27 | 1.57 | L | 2 | 1;4 |  |
| RR_C16_S31.40_G | chr16p11.2-q12.1 | 16 | 31.40 | 45.52 | 14.12 | G | 2 | 3;4 |  |
| RR_C16_S54.25_G | chr16q12.2-q22.1 | 16 | 54.25 | 69.11 | 14.87 | G | 4 | 1;2;3;4 | *POLR2C, KIFC3, CSNK2A2, GOT2, CDH5, PSKH1, PSMB10, VPS4A,* |
| RR_C16_S70.23_G | chr16q22.3-q24.3 | 16 | 70.23 | 80.69 | 10.46 | G | 3 | 2;3;4 | *PSMD7,PLCG2* |
| RR_C16_S82.56_G | chr16q22.3-q24.3 | 16 | 82.56 | 88.62 | 6.06 | G | 4 | 1;2;3;4 | *GALNS* |
| RR_C17_S1.27_L | chr17p13.3 | 17 | 1.27 | 1.91 | 0.64 | L | 2 | 2;4 |  |
| RR_C17_S22.65_G | chr17q11.1-q11.2 | 17 | 22.65 | 24.43 | 1.78 | G | 2 | 2;4 | *SPAG5* |
| RR_C17_S34.18_G | chr17q12-q21.2 | 17 | 34.18 | 35.91 | 1.74 | G | 3 | 2;3;4 | *CDC6,RARA,ERBB2* |
| RR_C17_S53.73_G | chr17q22-q23.3 | 17 | 53.73 | 59.27 | 5.54 | G | 3 | 1;3;4 | *DDX42,PSMC5* |
| RR_C17_S61.06_G | chr17q24.1-q24.2 | 17 | 61.06 | 63.17 | 2.11 | G | 3 | 1;3;4 | *PRKCA* |
| RR_C17_S68.79_G | chr17q25.1-q25.3 | 17 | 68.79 | 74.29 | 5.50 | G | 2 | 1;4 | *GRB2,CDK3,SPHK1,BIRC5* |
| RR_C17_S75.38_G | chr17q25.3 | 17 | 75.38 | 78.60 | 3.22 | G | 3 | 2;3;4 | *BAIAP2,AATK* |
| RR_C18_S5.23_L | chr18p11.31-p11.22 | 18 | 5.23 | 10.54 | 5.31 | L | 2 | 2;4 | *RALBP1,RAB31* |
| RR_C19_S12.65_G | chr19p13.13-p13.12 | 19 | 12.65 | 12.85 | 0.20 | G | 2 | 2;4 | *JUNB* |
| RR_C19_S13.75_G | chr19p13.13-p13.12 | 19 | 13.75 | 14.38 | 0.63 | G | 2 | 1;4 |  |
| RR_C19_S17.88_G | chr19p13.11-p12 | 19 | 17.88 | 19.48 | 1.60 | G | 3 | 1;2;4 | *MAST3, UPF1, DDX49* |
| RR_C19_S19.60_L | chr19p13.11-p12 | 19 | 19.60 | 22.07 | 2.46 | L | 2 | 3;4 |  |
| RR_C19_S40.21_G | chr19q13.11-q13.12 | 19 | 40.21 | 40.82 | 0.61 | G | 2 | 1;4 |  |
| RR_C19_S51.49_G | chr19q13.32 | 19 | 51.49 | 51.94 | 0.45 | G | 3 | 1;2;4 | *PTGIR* |
| RR_C19_S54.68_G | chr19q13.33-q13.42 | 19 | 54.68 | 55.24 | 0.56 | G | 3 | 1;2;4 |  |
| RR_C19_S57.59_G | chr19q13.33-q13.42 | 19 | 57.59 | 59.31 | 1.72 | G | 3 | 1;2;4 |  |
| RR_C19_S60.17_G | chr19q13.42-q13.43 | 19 | 60.17 | 62.65 | 2.48 | G | 3 | 1;2;4 |  |
| RR_C20_S0.071_G | chr20p13 | 20 | 0.07 | 1.32 | 1.25 | G | 2 | 2;4 |  |
| RR_C20_S3.14_G | chr20p13 | 20 | 3.14 | 3.75 | 0.62 | G | 2 | 2;4 |  |
| RR_C20_S28.24_G | chr20q11.1-q11.23 | 20 | 28.24 | 36.99 | 8.75 | G | 4 | 1;2;3;4 | *TPX2, APBA2BP, E2F1, EIF2S2, AHCY, GSS, PROCR, RBM39, SCAND1, DLGAP4, GHRH, BLCAP* |
| RR_C20_S44.43_G | chr20q13.12-q13.13 | 20 | 44.43 | 49.01 | 4.58 | G | 4 | 1;2;3;4 | *ARFGEF2,CSE1L* |
| RR_C20_S51.62_G | chr20q13.2-q13.33 | 20 | 51.62 | 54.65 | 3.03 | G | 2 | 1;4 | *AURKA, C20ORF43* |
| RR_C20_S55.60_G | chr20q13.2-q13.33 | 20 | 55.60 | 56.73 | 1.12 | G | 2 | 3;4 |  |
| RR_C20_S57.87_G | chr20q13.2-q13.33 | 20 | 57.87 | 62.06 | 4.19 | G | 3 | 1;3;4 | *PSMA7,RPS21, DIDO1, ARFRP1,ZGPAT* |
| RR_C21_S26.03_L | chr21q21.3 | 21 | 26.03 | 29.35 | 3.32 | L | 2 | 1;4 |  |
| RR_C21_S42.03_G | chr21q22.3 | 21 | 42.03 | 45.76 | 3.73 | G | 2 | 2;4 | *RIPK4, CBS* |
| RR_C22_S17.41_G | chr22q11.21 | 22 | 17.41 | 18.09 | 0.67 | G | 2 | 2;4 |  |
| RR_C23_S47.88_G | chr23p11.23 | 23 | 47.88 | 49.35 | 1.46 | G | 2 | 2;4 | *CACNA1F* |
| RR_C23_S53.13_G | chr23p11.22-p11.21 | 23 | 53.13 | 54.86 | 1.73 | G | 2 | 2;4 |  |
| RR_C23_S152.28_G | chr23q28 | 23 | 152.28 | 152.90 | 0.62 | G | 3 | 1;2;4 | *ATP6AP1* |
| RR_C23_S153.15_G | chr23q28 | 23 | 153.15 | 153.45 | 0.29 | G | 2 | 1;4 |  |

Table S2. CNV recurrent regions identified by GACE

| Ch | Start (Mb) | End (Mb) | Gain/Loss | Size (Mb) | Dataset Number | Dataset |
| --- | --- | --- | --- | --- | --- | --- |
| 1 | 150.65 | 152.02 | G | 1.38 | 3 | 2;4 |
| 1 | 153.29 | 154.10 | G | 0.81 | 4 | 1;3;4 |
| 1 | 208.92 | 235.13 | G | 26.21 | 4 | 1;2;4 |
| 2 | 3.36 | 9.99 | G | 6.63 | 3 | 2;4 |
| 2 | 71.41 | 74.67 | G | 3.26 | 3 | 1;4 |
| 2 | 216.73 | 220.15 | G | 3.42 | 3 | 2;4 |
| 3 | 158.64 | 168.58 | G | 9.94 | 4 | 1;3;4 |
| 3 | 180.35 | 188.01 | G | 7.66 | 5 | 1;2;3;4 |
| 4 | 0.94 | 3.01 | G | 2.07 | 4 | 1;2;4 |
| 5 | 0.27 | 10.32 | G | 10.05 | 3 | 1;4 |
| 5 | 175.10 | 179.71 | G | 4.62 | 5 | 1;2;3;4 |
| 6 | 26.13 | 28.23 | G | 2.11 | 4 | 1;3;4 |
| 6 | 33.35 | 37.56 | G | 4.21 | 4 | 1;2;4 |
| 6 | 41.76 | 44.33 | G | 2.57 | 3 | 1;4 |
| 7 | 0.06 | 6.41 | G | 6.35 | 4 | 1;2;4 |
| 7 | 71.05 | 74.86 | G | 3.81 | 4 | 1;2;4 |
| 8 | 95.72 | 108.58 | G | 12.86 | 4 | 1;3;4 |
| 8 | 140.81 | 146.25 | G | 5.44 | 5 | 1;2;3;4 |
| 9 | 138.82 | 139.30 | G | 0.47 | 3 | 2;4 |
| 10 | 133.60 | 135.22 | G | 1.63 | 3 | 2;4 |
| 11 | 63.08 | 64.28 | G | 1.21 | 3 | 2;4 |
| 11 | 64.61 | 71.82 | G | 7.22 | 4 | 1;2;4 |
| 12 | 121.52 | 122.52 | G | 1.00 | 4 | 1;3;4 |
| 12 | 130.88 | 131.92 | G | 1.04 | 3 | 1;4 |
| 13 | 49.55 | 69.58 | G | 20.03 | 3 | 1;2;3 |
| 14 | 32.48 | 35.35 | G | 2.87 | 3 | 3;4 |
| 15 | 38.48 | 39.58 | G | 1.10 | 3 | 2;4 |
| 16 | 56.04 | 65.82 | G | 9.78 | 4 | 2;3;4 |
| 16 | 66.25 | 69.82 | G | 3.57 | 4 | 2;3;4 |
| 16 | 84.39 | 88.62 | G | 4.23 | 4 | 1;2;4 |
| 17 | 23.39 | 24.94 | G | 1.55 | 3 | 2;4 |
| 17 | 53.73 | 59.30 | G | 5.57 | 4 | 1;3;4 |
| 17 | 69.78 | 75.38 | G | 5.59 | 4 | 1;2;4 |
| 17 | 75.76 | 78.60 | G | 2.84 | 3 | 2;4 |
| 19 | 40.51 | 41.87 | G | 1.35 | 3 | 3;4 |
| 19 | 54.78 | 55.61 | G | 0.84 | 4 | 1;2;4 |
| 19 | 60.28 | 62.76 | G | 2.49 | 3 | 1;2;3 |
| 20 | 33.51 | 35.01 | G | 1.51 | 3 | 2;4 |
| 20 | 59.98 | 62.16 | G | 2.17 | 4 | 1;3;4 |
| 21 | 43.35 | 46.25 | G | 2.90 | 3 | 1;4 |
| 22 | 17.28 | 19.27 | G | 1.99 | 5 | 1;2;3;4 |
| 23 | 47.88 | 49.53 | G | 1.65 | 3 | 2;4 |
| 23 | 152.43 | 152.83 | G | 0.39 | 3 | 1;4 |
| 23 | 153.15 | 153.43 | G | 0.28 | 3 | 1;4 |
| 1 | 64.98 | 78.38 | L | 13.39 | 4 | 1;2;4 |
| 1 | 88.92 | 108.54 | L | 19.62 | 3 | 2;4 |
| 2 | 170.26 | 173.84 | L | 3.58 | 3 | 1;4 |
| 2 | 178.98 | 191.27 | L | 12.28 | 4 | 1;2;4 |
| 4 | 156.35 | 164.49 | L | 8.14 | 3 | 2;4 |
| 5 | 37.85 | 43.52 | L | 5.67 | 3 | 1;2;3 |
| 5 | 54.31 | 57.79 | L | 3.48 | 3 | 3;4 |
| 5 | 149.36 | 150.93 | L | 1.57 | 3 | 1;2;3 |
| 6 | 116.68 | 132.95 | L | 16.27 | 3 | 2;4 |
| 7 | 86.97 | 94.12 | L | 7.15 | 3 | 2;4 |
| 7 | 104.54 | 116.75 | L | 12.21 | 4 | 1;2;4 |
| 8 | 9.79 | 31.15 | L | 21.36 | 5 | 1;2;3;4 |
| 9 | 0.46 | 10.19 | L | 9.73 | 3 | 2;4 |
| 9 | 93.37 | 97.12 | L | 3.75 | 4 | 1;2;4 |
| 10 | 44.11 | 48.04 | L | 3.92 | 5 | 1;2;3;4 |
| 11 | 94.35 | 104.38 | L | 10.04 | 3 | 2;4 |
| 11 | 119.85 | 124.81 | L | 4.96 | 4 | 1;3;4 |
| 11 | 128.75 | 133.63 | L | 4.88 | 3 | 2;4 |
| 12 | 6.93 | 10.48 | L | 3.55 | 3 | 1;2;3 |
| 12 | 21.81 | 27.74 | L | 5.93 | 4 | 1;2;4 |
| 16 | 9.76 | 22.84 | L | 13.07 | 5 | 1;2;3;4 |
| 16 | 30.49 | 32.69 | L | 2.20 | 3 | 1;2;3 |
| 17 | 0.85 | 2.56 | L | 1.71 | 4 | 1;2;4 |
| 17 | 7.44 | 19.82 | L | 12.39 | 4 | 1;2;4 |
| 17 | 35.85 | 36.98 | L | 1.13 | 3 | 3;4 |
| 20 | 3.99 | 24.89 | L | 20.90 | 3 | 1;4 |
| 21 | 18.20 | 29.66 | L | 11.46 | 3 | 1;4 |
| 23 | 100.49 | 102.56 | L | 2.07 | 3 | 2;4 |

Table S3 GO annotation of the local genes located on the recurrent regions either commonly identified by both ACE methods or uniquely by a specific method

| **Gain recurrent regions identified by both methods** | | | | | | |
| --- | --- | --- | --- | --- | --- | --- |
| PathName | hit.no | input.no | path.no | bgd.no | Fisher.p | fold.enriched |
| nucleosome assembly | 51 | 1215 | 74 | 13097 | 0.00E+00 | 7.43 |
| nucleosome organization | 53 | 1215 | 84 | 13097 | 0.00E+00 | 6.80 |
| chromatin assembly | 53 | 1215 | 84 | 13097 | 0.00E+00 | 6.80 |
| Systemic lupus erythematosus | 48 | 1215 | 101 | 13097 | 0.00E+00 | 5.12 |
| chromatin assembly or disassembly | 61 | 1215 | 129 | 13097 | 0.00E+00 | 5.10 |
| DNA packaging | 61 | 1215 | 131 | 13097 | 0.00E+00 | 5.02 |
| protein-DNA complex assembly | 52 | 1215 | 132 | 13097 | 0.00E+00 | 4.25 |
| Chromatin packaging and remodeling | 54 | 1215 | 160 | 13097 | 0.00E+00 | 3.64 |
| establishment or maintenance of chromatin architecture | 84 | 1215 | 351 | 13097 | 1.11E-16 | 2.58 |
| chromosome organization | 96 | 1215 | 476 | 13097 | 1.13E-13 | 2.17 |
| cellular macromolecular complex assembly | 83 | 1215 | 417 | 13097 | 1.16E-11 | 2.15 |
| Golgi organization | 9 | 1215 | 31 | 13097 | 1.52E-03 | 3.13 |
| vesicle organization | 11 | 1215 | 46 | 13097 | 2.66E-03 | 2.58 |
| Biosynthesis of steroids | 7 | 1215 | 23 | 13097 | 3.77E-03 | 3.28 |
| Anterior/posterior patterning | 9 | 1215 | 37 | 13097 | 5.64E-03 | 2.62 |
| vesicle coating | 6 | 1215 | 19 | 13097 | 5.93E-03 | 3.40 |
| rRNA metabolism | 11 | 1215 | 51 | 13097 | 6.20E-03 | 2.32 |
| membrane budding | 7 | 1215 | 25 | 13097 | 6.27E-03 | 3.02 |
| Segment specification | 13 | 1215 | 66 | 13097 | 6.88E-03 | 2.12 |
| regulation of chromosome organization | 10 | 1215 | 47 | 13097 | 9.79E-03 | 2.29 |
|  |  |  |  |  |  |  |
| **Deleted recurrent regions identified by both methods** | | | | | | |
| PathName | hit.no | input.no | path.no | bgd.no | Fisher.p | fold.enriched |
| cell surface receptor linked signal transduction | 48 | 229 | 1718 | 13097 | 5.86E-04 | 1.60 |
| cholesterol metabolic process | 7 | 229 | 96 | 13097 | 1.45E-03 | 4.17 |
| complement activation, classical pathway | 4 | 229 | 29 | 13097 | 1.53E-03 | 7.89 |
| cytolysis | 4 | 229 | 31 | 13097 | 1.98E-03 | 7.38 |
| humoral immune response mediated by circulating immunoglobulin | 4 | 229 | 32 | 13097 | 2.23E-03 | 7.15 |
| morphogenesis of an epithelium | 7 | 229 | 105 | 13097 | 2.43E-03 | 3.81 |
| regulation of epithelial cell proliferation | 5 | 229 | 54 | 13097 | 2.46E-03 | 5.30 |
| regulation of ossification | 6 | 229 | 80 | 13097 | 2.74E-03 | 4.29 |
| sterol metabolic process | 7 | 229 | 108 | 13097 | 2.85E-03 | 3.71 |
| epithelial cell proliferation | 5 | 229 | 58 | 13097 | 3.38E-03 | 4.93 |
| gamete generation | 12 | 229 | 276 | 13097 | 3.42E-03 | 2.49 |
| regulation of cell motion | 14 | 229 | 352 | 13097 | 3.63E-03 | 2.27 |
| transmembrane receptor protein serine/threonine kinase signaling pathway | 8 | 229 | 150 | 13097 | 4.86E-03 | 3.05 |
| negative regulation of cell motion | 7 | 229 | 120 | 13097 | 5.11E-03 | 3.34 |
| transforming growth factor beta receptor signaling pathway | 7 | 229 | 122 | 13097 | 5.58E-03 | 3.28 |
| Complement and coagulation cascades | 5 | 229 | 66 | 13097 | 5.88E-03 | 4.33 |
| sexual reproduction | 13 | 229 | 335 | 13097 | 6.11E-03 | 2.22 |
| Fertilization | 3 | 229 | 22 | 13097 | 6.36E-03 | 7.80 |
| regulation of bone remodeling | 6 | 229 | 96 | 13097 | 6.71E-03 | 3.57 |
| Biosynthesis of steroids | 3 | 229 | 23 | 13097 | 7.22E-03 | 7.46 |
| regulation of tissue remodeling | 6 | 229 | 99 | 13097 | 7.77E-03 | 3.47 |
| microtubule-based process | 11 | 229 | 275 | 13097 | 9.11E-03 | 2.29 |
| regulation of cell migration | 12 | 229 | 314 | 13097 | 9.30E-03 | 2.19 |
|  |  |  |  |  |  |  |
| **Gained recurrent regions uniquely identified by WACE** | | | | | |  |
| PathName | hit.no | input.no | path.no | bgd.no | Fisher.p | fold.enriched |
| cellular zinc ion homeostasis | 8 | 895 | 15 | 13097 | 1.93E-06 | 7.80 |
| response to metal ion | 18 | 895 | 120 | 13097 | 1.28E-03 | 2.20 |
| protein ubiquitination | 20 | 895 | 145 | 13097 | 2.03E-03 | 2.02 |
| G1 phase of mitotic cell cycle | 7 | 895 | 28 | 13097 | 2.26E-03 | 3.66 |
| response to inorganic substance | 19 | 895 | 137 | 13097 | 2.43E-03 | 2.03 |
| establishment of organelle localization | 12 | 895 | 72 | 13097 | 3.29E-03 | 2.44 |
| estrogen receptor signaling pathway | 7 | 895 | 30 | 13097 | 3.45E-03 | 3.41 |
| insulin receptor signaling pathway | 10 | 895 | 56 | 13097 | 4.23E-03 | 2.61 |
| regulation of S phase | 8 | 895 | 39 | 13097 | 4.24E-03 | 3.00 |
| cholesterol metabolic process | 14 | 895 | 96 | 13097 | 5.50E-03 | 2.13 |
| tyrosine phosphorylation of Stat3 protein | 6 | 895 | 25 | 13097 | 5.76E-03 | 3.51 |
| G1 phase | 7 | 895 | 33 | 13097 | 6.05E-03 | 3.10 |
| regulation of S phase of mitotic cell cycle | 7 | 895 | 34 | 13097 | 7.18E-03 | 3.01 |
| regulation of interleukin-6 production | 10 | 895 | 61 | 13097 | 7.88E-03 | 2.40 |
| Glioma | 10 | 895 | 61 | 13097 | 7.88E-03 | 2.40 |
| Non-small cell lung cancer | 9 | 895 | 53 | 13097 | 9.10E-03 | 2.48 |
| Phosphatidylinositol signaling system | 11 | 895 | 72 | 13097 | 9.30E-03 | 2.24 |
| Miscellaneous | 12 | 895 | 82 | 13097 | 9.50E-03 | 2.14 |
|  |  |  |  |  |  |  |
| **Deleted recurrent regions uniquely identified by WACE** | | |  |  |  |  |
| PathName | hit.no | input.no | path.no | bgd.no | Fisher.p | fold.enriched |
| MHCII-mediated immunity | 9 | 213 | 19 | 13097 | 5.39E-12 | 29.13 |
| antigen processing and presentation | 12 | 213 | 69 | 13097 | 9.81E-10 | 10.69 |
| Asthma | 8 | 213 | 24 | 13097 | 2.52E-09 | 20.50 |
| Allograft rejection | 8 | 213 | 29 | 13097 | 1.37E-08 | 16.96 |
| Graft-versus-host disease | 8 | 213 | 30 | 13097 | 1.85E-08 | 16.40 |
| Antigen processing and presentation | 11 | 213 | 72 | 13097 | 2.06E-08 | 9.39 |
| Autoimmune thyroid disease | 9 | 213 | 44 | 13097 | 2.91E-08 | 12.58 |
| Type I diabetes mellitus | 8 | 213 | 35 | 13097 | 6.92E-08 | 14.05 |
| Cell adhesion molecules (CAMs) | 11 | 213 | 113 | 13097 | 2.22E-06 | 5.99 |
| antigen processing and presentation of peptide antigen | 5 | 213 | 28 | 13097 | 7.86E-05 | 10.98 |
| Systemic lupus erythematosus | 8 | 213 | 101 | 13097 | 2.37E-04 | 4.87 |
| T-cell mediated immunity | 9 | 213 | 148 | 13097 | 7.00E-04 | 3.74 |
| Notch signaling pathway | 5 | 213 | 51 | 13097 | 1.39E-03 | 6.03 |
| positive regulation of transcription from RNA polymerase II promoter | 10 | 213 | 221 | 13097 | 3.34E-03 | 2.78 |
| Notch signaling pathway | 4 | 213 | 39 | 13097 | 3.58E-03 | 6.31 |
| Pancreatic cancer | 5 | 213 | 72 | 13097 | 6.29E-03 | 4.27 |
| aging | 6 | 213 | 105 | 13097 | 7.31E-03 | 3.51 |
| response to heat | 5 | 213 | 77 | 13097 | 8.32E-03 | 3.99 |
|  |  |  |  |  |  |  |
| **Gained recurrent regions uniquely identified by GACE** | | | | | |  |
| PathName | hit.no | input.no | path.no | bgd.no | Fisher.p | fold.enriched |
| epidermis morphogenesis | 7 | 415 | 40 | 13097 | 2.30E-04 | 5.52 |
| epidermal cell differentiation | 6 | 415 | 30 | 13097 | 3.04E-04 | 6.31 |
| regulation of Rho GTPase activity | 5 | 415 | 31 | 13097 | 2.68E-03 | 5.09 |
| tissue morphogenesis | 7 | 415 | 63 | 13097 | 3.66E-03 | 3.51 |
| regulation of Ras GTPase activity | 6 | 415 | 51 | 13097 | 5.27E-03 | 3.71 |
| positive regulation of B cell activation | 4 | 415 | 23 | 13097 | 5.45E-03 | 5.49 |
| positive regulation of Ras GTPase activity | 4 | 415 | 24 | 13097 | 6.38E-03 | 5.26 |
| protein refolding | 4 | 415 | 26 | 13097 | 8.55E-03 | 4.86 |
|  |  |  |  |  |  |  |
| **Deleted recurrent regions uniquely identified by GACE** | | | | |  |  |
| PathName | hit.no | input.no | path.no | bgd.no | Fisher.p | fold.enriched |
| Cell Communication | 26 | 724 | 113 | 13097 | 3.51E-10 | 4.16 |
| collagen metabolic process | 12 | 724 | 37 | 13097 | 3.83E-07 | 5.87 |
| collagen catabolic process | 10 | 724 | 26 | 13097 | 5.93E-07 | 6.96 |
| multicellular organismal macromolecule metabolic process | 12 | 724 | 39 | 13097 | 7.30E-07 | 5.57 |
| multicellular organismal metabolic process | 12 | 724 | 43 | 13097 | 2.33E-06 | 5.05 |
| multicellular organismal catabolic process | 10 | 724 | 30 | 13097 | 2.74E-06 | 6.03 |
| Natural killer cell mediated immunity | 10 | 724 | 47 | 13097 | 2.01E-04 | 3.85 |
| epidermis development | 20 | 724 | 163 | 13097 | 6.67E-04 | 2.22 |
| Muscle development | 14 | 724 | 104 | 13097 | 1.71E-03 | 2.44 |
| antimicrobial humoral response | 12 | 724 | 82 | 13097 | 1.74E-03 | 2.65 |
| ectoderm development | 20 | 724 | 179 | 13097 | 2.12E-03 | 2.02 |
| Tight junction | 15 | 724 | 120 | 13097 | 2.51E-03 | 2.26 |
| Gap junction | 12 | 724 | 91 | 13097 | 4.23E-03 | 2.39 |
| proteoglycan metabolic process | 8 | 724 | 49 | 13097 | 5.05E-03 | 2.95 |
| amino acid catabolic process | 8 | 724 | 49 | 13097 | 5.05E-03 | 2.95 |
| nucleoside triphosphate catabolic process | 5 | 724 | 22 | 13097 | 6.11E-03 | 4.11 |
| Cytokinesis | 10 | 724 | 73 | 13097 | 6.62E-03 | 2.48 |
| beta-Alanine metabolism | 5 | 724 | 23 | 13097 | 7.45E-03 | 3.93 |
| Valine, leucine and isoleucine degradation | 7 | 724 | 42 | 13097 | 7.60E-03 | 3.01 |
| Long-term depression | 10 | 724 | 75 | 13097 | 8.01E-03 | 2.41 |
| proteoglycan biosynthetic process | 6 | 724 | 34 | 13097 | 9.97E-03 | 3.19 |

Table S5 Known and novel breast cancer genes that are identified on the recurrent regions of amplification and validated by the siRNA experiments on multiple breast cancer cell lines.

1. Strang, G.N., Truong, *Wavelets and Filter Banks*. 1996, Wellesley, MA, USA: Wellesley-Cambrage Press.

2. Blaschke, F., et al., *Obesity, Peroxisome Proliferator-Activated Receptor, and Atherosclerosis in Type 2 Diabetes.* Arterioscler Thromb Vasc Biol, 2005.

3. Klevecz, R.R. and D.B. Murray, *Genome wide oscillations in expression. Wavelet analysis of time series data from yeast expression arrays uncovers the dynamic architecture of phenotype.* Mol Biol Rep, 2001. **28**(2): p. 73-82.

4. Moesa, H.A., C.D. K, and T. Akutsu, *Efficient determination of cluster boundaries for analysis of gene expression profile data using hierarchical clustering and wavelet transform.* Genome Inform, 2005. **16**(1): p. 132-41.

5. Andre, F., et al., *Molecular characterization of breast cancer with high-resolution oligonucleotide comparative genomic hybridization array.* Clin Cancer Res, 2009. **15**(2): p. 441-51.

6. Fridlyand, J., et al., *Breast tumor copy number aberration phenotypes and genomic instability.* BMC Cancer, 2006. **6**: p. 96.

7. Pollack, J.R., et al., *Microarray analysis reveals a major direct role of DNA copy number alteration in the transcriptional program of human breast tumors.* Proc Natl Acad Sci U S A, 2002. **99**(20): p. 12963-8.

8. Haverty, P.M., et al., *High-resolution genomic and expression analyses of copy number alterations in breast tumors.* Genes Chromosomes Cancer, 2008. **47**(6): p. 530-42.

9. Hu, G., et al., *MTDH activation by 8q22 genomic gain promotes chemoresistance and metastasis of poor-prognosis breast cancer.* Cancer Cell, 2009. **15**(1): p. 9-20.

10. Bergamaschi, A., et al., *Distinct patterns of DNA copy number alteration are associated with different clinicopathological features and gene-expression subtypes of breast cancer.* Genes Chromosomes Cancer, 2006. **45**(11): p. 1033-40.

11. Zhu, J., et al., *Increasing the Power to Detect Causal Associations by Combining Genotypic and Expression Data in Segregating Populations.* PLoS Comput Biol, 2007. **3**(4): p. e69.

12. Zhang, B. and S. Horvath, *A general framework for weighted gene co-expression network analysis.* Stat Appl Genet Mol Biol, 2005. **4**: p. Article17.

13. Okamura, H., et al., *Histone H1.2 is translocated to mitochondria and associates with Bak in bleomycin-induced apoptotic cells.* J Cell Biochem, 2008. **103**(5): p. 1488-96.

14. Fechter, A., et al., *Cloning of genetically tagged chromosome break sequences reveals new fragile sites at 6p21 and 13q22.* Int J Cancer, 2007. **120**(11): p. 2359-67.

15. Maleno, I., et al., *Distribution of HLA class I altered phenotypes in colorectal carcinomas: high frequency of HLA haplotype loss associated with loss of heterozygosity in chromosome region 6p21.* Immunogenetics, 2004. **56**(4): p. 244-53.

16. Hwang, K.T., et al., *Genomic copy number alterations as predictive markers of systemic recurrence in breast cancer.* Int J Cancer, 2008. **123**(8): p. 1807-15.

17. Mootha, V.K., et al., *PGC-1alpha-responsive genes involved in oxidative phosphorylation are coordinately downregulated in human diabetes.* Nat Genet, 2003. **34**(3): p. 267-73.

18. Ren, J.G., C. Jie, and C. Talbot, *How PEDF prevents angiogenesis: a hypothesized pathway.* Med Hypotheses, 2005. **64**(1): p. 74-8.

19. Zhang, B., et al., *Requirement for chromatin-remodeling complex in novel tumor suppressor HIC1-mediated transcriptional repression and growth control.* Oncogene, 2009. **28**(5): p. 651-61.

20. Parrella, P., et al., *HIC1 promoter methylation and 17p13.3 allelic loss in invasive ductal carcinoma of the breast.* Cancer Lett, 2005. **222**(1): p. 75-81.

21. Ishihara, T., et al., *ITCH is a putative target for a novel 20q11.22 amplification detected in anaplastic thyroid carcinoma cells by array-based comparative genomic hybridization.* Cancer Sci, 2008. **99**(10): p. 1940-9.

22. Scotto, L., et al., *Identification of copy number gain and overexpressed genes on chromosome arm 20q by an integrative genomic approach in cervical cancer: potential role in progression.* Genes Chromosomes Cancer, 2008. **47**(9): p. 755-65.

23. Wrage, M., et al., *Genomic profiles associated with early micrometastasis in lung cancer: relevance of 4q deletion.* Clin Cancer Res, 2009. **15**(5): p. 1566-74.

24. Heaney, J.D., et al., *Deletion of eIF2beta suppresses testicular cancer incidence and causes recessive lethality in agouti-yellow mice.* Hum Mol Genet, 2009.

(2009).
