# Supplementary material for: Inferring causal genomic alterations in breast cancer using gene expression data
Source: BMC Syst Biol. 2011 Aug 1;5:121. doi: 10.1186/1752-0509-5-121 (PMC3162519; doi:10.1186/1752-0509-5-121)
Supplement: Additional file 2 — Supplementary Table S4. siRNA screen signatures and the associated viability scores. [file 1752-0509-5-121-S2.PDF]

Table S4 siRNA screen signatures from all the four cell lines (ALL) and the individual cell lines (MCF7, MDA-MB-231, MDA-MB-468 and ZR-75-1). The signature from all the four cell lines is referred as S1 while the rest form S2, as described in the main text. The viability scores for the genes in S1 are the corresponding median values while the scores for the genes in S2 are the corresponding mean values.

Gene	Viability	Cell Line(s)	Signature Category
AATK	39.6	ALL	S1
ABCE1	35.8	ALL	S1
ABL2	30.2	ALL	S1
ACAA1	35.7	ALL	S1
ACADVL	35.9	ALL	S1
ADCK2	40.9	ALL	S1
AK1	39.4	ALL	S1
AKT2	29.6	ALL	S1
ALDH18A1	39.6	ALL	S1
ALDOA	35.6	ALL	S1
ARAF	32.4	ALL	S1
ARCN1	22.7	ALL	S1
ASCC3	39.7	ALL	S1
ASNS	35.6	ALL	S1
ATP6AP1	33.2	ALL	S1
ATP6V1C1	40.4	ALL	S1
AURKB	37.1	ALL	S1
BIRC5	35.9	ALL	S1
BLCAP	40	ALL	S1
BLMH	41.3	ALL	S1
BLVRA	28	ALL	S1
BRSK1	39.7	ALL	S1
C20orf52	27.8	ALL	S1
CAMKK2	38.6	ALL	S1
CARS	41.1	ALL	S1
CBS	33.4	ALL	S1
CCNA2	33.5	ALL	S1
CCNB3	31.8	ALL	S1
CD47	14.2	ALL	S1
CDC2	31.6	ALL	S1
CDC27	29.1	ALL	S1
CDC5L	29.9	ALL	S1
CDC6	33	ALL	S1
CDH5	34.8	ALL	S1
CDKL2	40	ALL	S1
CFLAR	21.6	ALL	S1
CHEK2	35.1	ALL	S1
CNOT6	37.1	ALL	S1
COX6A1	39.3	ALL	S1
CPT1A	29.9	ALL	S1
CSE1L	40.9	ALL	S1

CSNK1A1	38.4	ALL	S1
CTDP1	32.3	ALL	S1
DAPK2	33.5	ALL	S1
DCAMKL1	32.7	ALL	S1
DCC	38.9	ALL	S1
DDX41	38.3	ALL	S1
DDX42	39.3	ALL	S1
DDX48	12.9	ALL	S1
DHX8	25.3	ALL	S1
DLGAP4	38.6	ALL	S1
DSG2	31.3	ALL	S1
DTYMK	35.1	ALL	S1
DYRK4	34.4	ALL	S1
ECT2	22.2	ALL	S1
EGR1	40	ALL	S1
EIF2S2	37.2	ALL	S1
ENO3	34.3	ALL	S1
EPHB6	37.6	ALL	S1
ERCC2	41.3	ALL	S1
ERCC6	39.8	ALL	S1
FBXL10	35.7	ALL	S1
FBXO10	24.3	ALL	S1
FBXW4	26.3	ALL	S1
FDPS	39.6	ALL	S1
FES	37.4	ALL	S1
FLJ20035	29.5	ALL	S1
FLJ40852	33.8	ALL	S1
FNBP3	35.1	ALL	S1
FOS	39.6	ALL	S1
FZD2	36.6	ALL	S1
FZD4	40.2	ALL	S1
GALNS	36.9	ALL	S1
GBA	31.3	ALL	S1
GMEB2	21.8	ALL	S1
GNAI1	29.1	ALL	S1
GNAS	38.2	ALL	S1
GOT2	34.8	ALL	S1
GPR125	35.4	ALL	S1
GRAP	20.7	ALL	S1
GSG2	25.8	ALL	S1
GUCY1B3	19.8	ALL	S1
GUCY2D	29.7	ALL	S1
HCN3	29.1	ALL	S1
HCN4	28.3	ALL	S1
HRAS	39.9	ALL	S1
HSD17B4	31.6	ALL	S1
HSPB1	37.2	ALL	S1

IMPDH2	24.7	ALL	S1
IRS1	38.8	ALL	S1
JUNB	39	ALL	S1
KALRN	40.3	ALL	S1
KCNC3	33.5	ALL	S1
KCNC4	33.4	ALL	S1
KCND3	23.8	ALL	S1
KCNG1	41.2	ALL	S1
KIAA0368	39.2	ALL	S1
KIF11	18.8	ALL	S1
KIF18A	39.6	ALL	S1
KIF26B	37.1	ALL	S1
KIF2C	27.2	ALL	S1
KNTC2	25.7	ALL	S1
LOC12652(	31	ALL	S1
LRP5	39	ALL	S1
MAD2L1	31	ALL	S1
MAP3K7	40.2	ALL	S1
MAPK13	38.2	ALL	S1
MAPK7	27.2	ALL	S1
MAPKAPK2	33.9	ALL	S1
MARK4	35.9	ALL	S1
MAST3	25.3	ALL	S1
MEN1	30.8	ALL	S1
MINK1	40.4	ALL	S1
MUSK	41.3	ALL	S1
MYD88	38.9	ALL	S1
MYLK	36.1	ALL	S1
MYO5A	27.4	ALL	S1
NCOA5	37.1	ALL	S1
NET1	36.5	ALL	S1
NOTCH1	41.1	ALL	S1
NR1I3	21.1	ALL	S1
NR2F1	32.9	ALL	S1
NUAK2	34.9	ALL	S1
NUP153	40.5	ALL	S1
PAK2	40.2	ALL	S1
PAK7	37.2	ALL	S1
PDE6B	40.6	ALL	S1
PDK2	23.4	ALL	S1
PGD	38.9	ALL	S1
PHKG1	40.7	ALL	S1
PIK3CA	33.8	ALL	S1
PIP5K1A	26.2	ALL	S1
PIWIL1	41	ALL	S1
PLCG1	37.4	ALL	S1
PLK1	20.1	ALL	S1

PLK3	36	ALL	S1
PLOD3	36.5	ALL	S1
POLR2A	6.9	ALL	S1
POLR2B	36.2	ALL	S1
POLR2C	39.3	ALL	S1
POLR2D	39.7	ALL	S1
POLR2E	38	ALL	S1
POLR2F	18.2	ALL	S1
POLR2G	31.6	ALL	S1
PPP1CC	32.6	ALL	S1
PRKCD	31.5	ALL	S1
PRKY	32.6	ALL	S1
PROCR	26.6	ALL	S1
PSMA2	35.6	ALL	S1
PSMA3	32	ALL	S1
PSMA4	30	ALL	S1
PSMA7	36.1	ALL	S1
PSMB1	32.5	ALL	S1
PSMB10	31.6	ALL	S1
PSMB2	41.1	ALL	S1
PSMB3	24.3	ALL	S1
PSMB4	26.4	ALL	S1
PSMB6	38.2	ALL	S1
PSMB7	38.2	ALL	S1
PSMC1	38.1	ALL	S1
PSMC2	28.7	ALL	S1
PSMC3	29.2	ALL	S1
PSMC4	27.2	ALL	S1
PSMC5	41	ALL	S1
PSMC6	32	ALL	S1
PSMD2	29.4	ALL	S1
PSMD7	31.6	ALL	S1
PSMD8	14.6	ALL	S1
PTEN	36.4	ALL	S1
PTGIR	34.4	ALL	S1
PTK2	39	ALL	S1
PTP4A2	33.3	ALL	S1
PTPN23	36.8	ALL	S1
PTPRA	29.8	ALL	S1
PTPRK	33.9	ALL	S1
PTPRU	28.2	ALL	S1
RALBP1	30.7	ALL	S1
RARA	20.2	ALL	S1
RENT1	41.2	ALL	S1
RIOK1	30.1	ALL	S1
RIPK4	39.8	ALL	S1
RNPC2	31.2	ALL	S1

RRM1	17.8	ALL	S1
RRM2	25	ALL	S1
SCAND1	36.6	ALL	S1
SCN4A	39.6	ALL	S1
SCYL2	40.6	ALL	S1
SFRS10	32.3	ALL	S1
SLC31A1	33	ALL	S1
SLC39A10	31.3	ALL	S1
SMPD2	33.2	ALL	S1
SPAG5	33	ALL	S1
STK11	33.9	ALL	S1
STK25	35.7	ALL	S1
STK40	34.9	ALL	S1
STK6	39.6	ALL	S1
STMN1	41.2	ALL	S1
TACC3	40.2	ALL	S1
TERF2	40.8	ALL	S1
TFRC	39.5	ALL	S1
THOC2	36.2	ALL	S1
TKT	36.2	ALL	S1
TOP1	39.6	ALL	S1
TPX2	36.1	ALL	S1
TRPM3	38.4	ALL	S1
TSC1	36.1	ALL	S1
TSSK4	34.5	ALL	S1
UBE2D4	40.8	ALL	S1
UBE2I	40.1	ALL	S1
UROS	40.6	ALL	S1
VCP	19.6	ALL	S1
VPS4B	35.8	ALL	S1
WEE1	33.9	ALL	S1
YES1	40.4	ALL	S1
ZA20D1	31.8	ALL	S1
ZGPAT	34.6	ALL	S1
ABCE1	37.11	MCF7	S2
ACAA1	39.04	MCF7	S2
ACADVL	28.6	MCF7	S2
ACVR2B	38.68	MCF7	S2
ADAR	30.4	MCF7	S2
ADCK5	34.92	MCF7	S2
AKT2	35.08	MCF7	S2
AKT3	33.06	MCF7	S2
ALDH18A1	36.8	MCF7	S2
ALS2CR2	33.37	MCF7	S2
AR	35.91	MCF7	S2
ARCN1	36.05	MCF7	S2
ARL1	38.23	MCF7	S2

ASNS	39.63	MCF7	S2
ATP6AP1	38.69	MCF7	S2
ATP6V1B2	35.38	MCF7	S2
ATP6V1C1	37.97	MCF7	S2
BCR	40.55	MCF7	S2
BIRC7	34.16	MCF7	S2
BLCAP	35.42	MCF7	S2
CAPN2	38.35	MCF7	S2
CARS	36.68	MCF7	S2
CD38	39.85	MCF7	S2
CD47	26.16	MCF7	S2
CDC2	34.29	MCF7	S2
CDC42BPG	39.97	MCF7	S2
CDC5L	25.77	MCF7	S2
CDH5	35.56	MCF7	S2
CFLAR	24.98	MCF7	S2
CHEK1	39.22	MCF7	S2
CHN1	39.64	MCF7	S2
CNOT6	37.55	MCF7	S2
COL9A3	34.65	MCF7	S2
CTBP1	37.44	MCF7	S2
CTDP1	27.63	MCF7	S2
CUL3	35.86	MCF7	S2
DAPK2	37.71	MCF7	S2
DCAMKL1	40.96	MCF7	S2
DCC	38.12	MCF7	S2
DDX18	39.54	MCF7	S2
DDX48	19.79	MCF7	S2
DHX8	29.39	MCF7	S2
DLGAP4	36.07	MCF7	S2
DSG2	29.52	MCF7	S2
DTYMK	33.82	MCF7	S2
DVL2	24.88	MCF7	S2
DYRK3	40.54	MCF7	S2
ECT2	27.57	MCF7	S2
EIF2S2	37.4	MCF7	S2
ENO3	30.83	MCF7	S2
EPHB6	35.47	MCF7	S2
ERCC6	25.34	MCF7	S2
FASTK	38.22	MCF7	S2
FBXL10	37.25	MCF7	S2
FBXW4	24.5	MCF7	S2
FDPS	30.47	MCF7	S2
FGFR2	39.42	MCF7	S2
FLJ40852	34.63	MCF7	S2
FNBP3	30.94	MCF7	S2
FZD4	38.84	MCF7	S2

GALE	40	MCF7	S2
GBA	32.15	MCF7	S2
GMEB2	25.08	MCF7	S2
GNAI1	25.36	MCF7	S2
GNAS	31.89	MCF7	S2
GOT2	29.83	MCF7	S2
GPSM2	40.47	MCF7	S2
GRAP	24.54	MCF7	S2
GRK6	34.5	MCF7	S2
GSTM4	40.55	MCF7	S2
GUCY1B3	34.27	MCF7	S2
GUCY2D	28.1	MCF7	S2
GUK1	40.66	MCF7	S2
HACE1	38.35	MCF7	S2
HCN3	30.08	MCF7	S2
HCN4	20.24	MCF7	S2
HEATR1	36.59	MCF7	S2
HLA-A	37.7	MCF7	S2
HMMR	32.14	MCF7	S2
HMOX2	34.71	MCF7	S2
HSD17B4	29.3	MCF7	S2
HSPB1	38.31	MCF7	S2
IER3	40.62	MCF7	S2
IGF1R	38.48	MCF7	S2
IKBKE	39.58	MCF7	S2
IMPDH2	37.96	MCF7	S2
IRAK1	39.01	MCF7	S2
IRS1	29.85	MCF7	S2
JUNB	34.45	MCF7	S2
KALRN	30.99	MCF7	S2
KCNC3	32.01	MCF7	S2
KCNC4	30.42	MCF7	S2
KCND3	18.85	MCF7	S2
KIF11	22.46	MCF7	S2
KIF22	33.61	MCF7	S2
KIF26B	34.27	MCF7	S2
KIFC1	38.82	MCF7	S2
KIFC2	39.07	MCF7	S2
KIFC3	40.42	MCF7	S2
KIT	33.78	MCF7	S2
KNTC2	30.34	MCF7	S2
LOC12652(	39.37	MCF7	S2
MAD2L1	36.57	MCF7	S2
MAD2L2	34.82	MCF7	S2
MAP3K12	38.9	MCF7	S2
MAP3K4	36.19	MCF7	S2
MAPK13	30.71	MCF7	S2

MAPK15	30.77	MCF7	S2
MAPK4	37.12	MCF7	S2
MAPK7	30.25	MCF7	S2
MARK4	33.16	MCF7	S2
MAST1	23.05	MCF7	S2
MAST3	36.79	MCF7	S2
MCL1	29.27	MCF7	S2
MDM2	33.62	MCF7	S2
MEN1	39.84	MCF7	S2
MGST2	35.42	MCF7	S2
MNAT1	27.84	MCF7	S2
MUT	35.17	MCF7	S2
NEK3	33.25	MCF7	S2
NRBP2	38.81	MCF7	S2
NUAK2	40.71	MCF7	S2
NUP153	38.73	MCF7	S2
OAZ1	39.47	MCF7	S2
PAK7	37.66	MCF7	S2
PCTK3	30.75	MCF7	S2
PDGFRB	39.31	MCF7	S2
PDK2	38.25	MCF7	S2
PGM1	32.05	MCF7	S2
PHKA2	37.94	MCF7	S2
PIK3CA	24.32	MCF7	S2
PLCG1	30.04	MCF7	S2
PLK1	20.37	MCF7	S2
PLK3	39.02	MCF7	S2
POLR2A	10.61	MCF7	S2
POLR2B	26.72	MCF7	S2
POLR2C	32.86	MCF7	S2
POLR2E	34.93	MCF7	S2
POLR2F	22.28	MCF7	S2
POLR2G	31.19	MCF7	S2
POLR2H	36.54	MCF7	S2
POLR2K	40.82	MCF7	S2
POLR2L	36.63	MCF7	S2
PPP1CC	34.96	MCF7	S2
PPP2CA	38.69	MCF7	S2
PRKCA	31.63	MCF7	S2
PRKCD	40.95	MCF7	S2
PRKY	28.31	MCF7	S2
PROCR	40	MCF7	S2
PRPF4B	34.12	MCF7	S2
PSMB1	33.78	MCF7	S2
PSMB10	35.98	MCF7	S2
PSMB4	21.56	MCF7	S2
PSMB7	27.88	MCF7	S2

PSMC5	40.57	MCF7	S2
PSMD8	22.66	MCF7	S2
PTK2	36.52	MCF7	S2
PTPN22	39.73	MCF7	S2
PTPRU	29.19	MCF7	S2
RALBP1	28.47	MCF7	S2
ROCK1	32.38	MCF7	S2
RRM1	29.93	MCF7	S2
RRM2	29.02	MCF7	S2
RYK	39.5	MCF7	S2
SCAND1	35.8	MCF7	S2
SCYL2	31.72	MCF7	S2
SENP6	35.94	MCF7	S2
SFRS10	22.36	MCF7	S2
SKP1A	30.93	MCF7	S2
SLC25A29	28.62	MCF7	S2
SLC31A1	39.34	MCF7	S2
SLC39A10	31.47	MCF7	S2
SMPD2	34.18	MCF7	S2
SNF1LK	37.62	MCF7	S2
STAT1	40.33	MCF7	S2
STAT2	34.13	MCF7	S2
STK11	27.55	MCF7	S2
STK16	27.31	MCF7	S2
STK31	35.91	MCF7	S2
STK6	37.65	MCF7	S2
TACC3	36.36	MCF7	S2
TAF1	28.7	MCF7	S2
TFRC	39.79	MCF7	S2
THOC2	40.96	MCF7	S2
TIE1	38.01	MCF7	S2
TPX2	36.13	MCF7	S2
TRPM3	37.7	MCF7	S2
TSSK1	24.38	MCF7	S2
TSSK3	29.99	MCF7	S2
TUBB6	31.31	MCF7	S2
UBE2I	34.41	MCF7	S2
UCP3	40.72	MCF7	S2
VCP	24.16	MCF7	S2
VRK1	36.55	MCF7	S2
WASL	39.33	MCF7	S2
WEE1	38.42	MCF7	S2
ZA20D1	29.92	MCF7	S2
AATK	33.29	MDA-MB-231	S2
ABL2	32.56	MDA-MB-231	S2
ACADVL	32.35	MDA-MB-231	S2
ADCK2	25.77	MDA-MB-231	S2

ADCY3	32.74	MDA-MB-231	S2
ADRM1	28.53	MDA-MB-231	S2
AKT2	31.4	MDA-MB-231	S2
ALDOA	13.88	MDA-MB-231	S2
ANAPC2	29.65	MDA-MB-231	S2
ARAF	13.89	MDA-MB-231	S2
ARCN1	21.24	MDA-MB-231	S2
ARFRP1	30.68	MDA-MB-231	S2
ARID4A	28.85	MDA-MB-231	S2
ATP5D	34.61	MDA-MB-231	S2
B2M	31.28	MDA-MB-231	S2
BCL2L1	5.69	MDA-MB-231	S2
BIRC5	29.22	MDA-MB-231	S2
BLVRA	29.75	MDA-MB-231	S2
C20orf43	25.95	MDA-MB-231	S2
C20orf52	32.74	MDA-MB-231	S2
CAMK2G	26.54	MDA-MB-231	S2
CAMKK2	30.8	MDA-MB-231	S2
CBS	26.67	MDA-MB-231	S2
CCNA2	19.66	MDA-MB-231	S2
CCNB3	29.14	MDA-MB-231	S2
CCNG1	30.49	MDA-MB-231	S2
CD47	10.54	MDA-MB-231	S2
CDC27	11.82	MDA-MB-231	S2
CDC6	28.72	MDA-MB-231	S2
CDKL2	33.77	MDA-MB-231	S2
CHEK2	27.33	MDA-MB-231	S2
CLCN7	31.64	MDA-MB-231	S2
CLK3	22.05	MDA-MB-231	S2
COX6A1	28.49	MDA-MB-231	S2
CPT1A	2.61	MDA-MB-231	S2
CRKRS	33.82	MDA-MB-231	S2
CSF3R	28.9	MDA-MB-231	S2
CTNNAL1	32.5	MDA-MB-231	S2
CTSB	34.72	MDA-MB-231	S2
DAPK2	29.32	MDA-MB-231	S2
DCAMKL1	23.19	MDA-MB-231	S2
DDX19A	27.7	MDA-MB-231	S2
DDX41	33.05	MDA-MB-231	S2
DDX42	17.85	MDA-MB-231	S2
DDX48	6.09	MDA-MB-231	S2
DDX6	33.96	MDA-MB-231	S2
DHX8	21.16	MDA-MB-231	S2
DLGAP4	27.59	MDA-MB-231	S2
DNCH1	33.71	MDA-MB-231	S2
DYRK4	32.87	MDA-MB-231	S2
ECT2	20.08	MDA-MB-231	S2

EPHB6	21.59	MDA-MB-231	S2
FBXO10	25.71	MDA-MB-231	S2
FBXW4	33.76	MDA-MB-231	S2
FES	32.04	MDA-MB-231	S2
FLJ25006	33.37	MDA-MB-231	S2
FLNB	34.02	MDA-MB-231	S2
FNBP3	32.07	MDA-MB-231	S2
FZD2	19.56	MDA-MB-231	S2
GALNS	32.73	MDA-MB-231	S2
GBA	30.44	MDA-MB-231	S2
GMEB2	14.3	MDA-MB-231	S2
GNAI1	32.82	MDA-MB-231	S2
GPR125	24.38	MDA-MB-231	S2
GRAP	16.77	MDA-MB-231	S2
GSG2	19.93	MDA-MB-231	S2
GUCY1B2	25.31	MDA-MB-231	S2
GUCY1B3	24.17	MDA-MB-231	S2
GUCY2D	22.34	MDA-MB-231	S2
HCN3	28.1	MDA-MB-231	S2
HCN4	29.17	MDA-MB-231	S2
HDAC7A	30.3	MDA-MB-231	S2
HMGCR	16.92	MDA-MB-231	S2
HMMR	34.09	MDA-MB-231	S2
HRAS	21.1	MDA-MB-231	S2
HSPA6	32.47	MDA-MB-231	S2
IFT52	33.27	MDA-MB-231	S2
JUNB	26.08	MDA-MB-231	S2
KCND3	14.23	MDA-MB-231	S2
KCNH2	34.7	MDA-MB-231	S2
KIF11	29.55	MDA-MB-231	S2
KIF20A	10.14	MDA-MB-231	S2
KIF21A	11.62	MDA-MB-231	S2
KIF2C	26.23	MDA-MB-231	S2
KIF3C	27.54	MDA-MB-231	S2
LOC12652(	23.45	MDA-MB-231	S2
MAD2L1	30.04	MDA-MB-231	S2
MAP2K2	21.14	MDA-MB-231	S2
MAP3K2	30.83	MDA-MB-231	S2
MAP4K5	29.82	MDA-MB-231	S2
MAPK1	19.31	MDA-MB-231	S2
MAPK15	14.95	MDA-MB-231	S2
MAPK7	21.97	MDA-MB-231	S2
MARK4	26.99	MDA-MB-231	S2
MAST3	12.8	MDA-MB-231	S2
MGC11266	33.55	MDA-MB-231	S2
MGC3413	18.29	MDA-MB-231	S2
MPP3	12.97	MDA-MB-231	S2

MYO5A	10.31	MDA-MB-231	S2
NEK5	29.81	MDA-MB-231	S2
NR1I3	16.1	MDA-MB-231	S2
NR2F1	11.95	MDA-MB-231	S2
NUAK2	34.69	MDA-MB-231	S2
PDK2	19.77	MDA-MB-231	S2
PIK3CA	21.8	MDA-MB-231	S2
PIP5K1A	28.25	MDA-MB-231	S2
PLCG1	25.21	MDA-MB-231	S2
PLEKHG4	29.01	MDA-MB-231	S2
PLK1	19.74	MDA-MB-231	S2
PMS2	10.89	MDA-MB-231	S2
POLE	16.03	MDA-MB-231	S2
POLR2A	3.19	MDA-MB-231	S2
POLR2F	14.03	MDA-MB-231	S2
POLR2J	32.27	MDA-MB-231	S2
PPP1CC	30.23	MDA-MB-231	S2
PREX1	24.87	MDA-MB-231	S2
PRKCD	21.95	MDA-MB-231	S2
PROCR	10.37	MDA-MB-231	S2
PRPF4B	27.94	MDA-MB-231	S2
PSKH1	25.86	MDA-MB-231	S2
PSMA3	25.16	MDA-MB-231	S2
PSMA4	25.91	MDA-MB-231	S2
PSMA7	33.42	MDA-MB-231	S2
PSMB1	31.17	MDA-MB-231	S2
PSMB10	25.71	MDA-MB-231	S2
PSMB3	29	MDA-MB-231	S2
PSMC2	18.69	MDA-MB-231	S2
PSMC3	29.57	MDA-MB-231	S2
PSMC4	24.79	MDA-MB-231	S2
PSMC6	21.31	MDA-MB-231	S2
PSMD2	31.9	MDA-MB-231	S2
PSMD7	18.82	MDA-MB-231	S2
PSMD8	6.47	MDA-MB-231	S2
PTEN	24.94	MDA-MB-231	S2
PTGIR	25.23	MDA-MB-231	S2
PTP4A2	17.66	MDA-MB-231	S2
PTPN23	33.69	MDA-MB-231	S2
PTPRA	19.44	MDA-MB-231	S2
PTPRK	31.44	MDA-MB-231	S2
PTPRU	27.29	MDA-MB-231	S2
RAF1	32.07	MDA-MB-231	S2
RALB	19.66	MDA-MB-231	S2
RARA	8.69	MDA-MB-231	S2
RIOK1	25.01	MDA-MB-231	S2
RRM1	7.06	MDA-MB-231	S2

RRM2	10.33	MDA-MB-231	S2
SFRS10	34.66	MDA-MB-231	S2
SLC22A5	27.07	MDA-MB-231	S2
SLC31A1	28.67	MDA-MB-231	S2
SPHK1	25.14	MDA-MB-231	S2
STAT3	33.21	MDA-MB-231	S2
STK40	26.54	MDA-MB-231	S2
TAZ	28.96	MDA-MB-231	S2
TBK1	8.2	MDA-MB-231	S2
THOC2	26.78	MDA-MB-231	S2
TOPBP1	23.9	MDA-MB-231	S2
TSC1	10.13	MDA-MB-231	S2
TSSK4	26.79	MDA-MB-231	S2
VPS4B	26.53	MDA-MB-231	S2
WEE1	10.36	MDA-MB-231	S2
WFDC2	32.58	MDA-MB-231	S2
WNK1	24.77	MDA-MB-231	S2
ZA20D1	25.95	MDA-MB-231	S2
ZGPAT	22.54	MDA-MB-231	S2
AATK	4.22	MDA-MB-468	S2
ABL2	27.82	MDA-MB-468	S2
ACAA1	19.13	MDA-MB-468	S2
ACADSB	14.86	MDA-MB-468	S2
AKT2	27.72	MDA-MB-468	S2
ALDOA	12.38	MDA-MB-468	S2
ANAPC5	24.33	MDA-MB-468	S2
ANLN	26.63	MDA-MB-468	S2
APBA2BP	24.9	MDA-MB-468	S2
ARAF	18.05	MDA-MB-468	S2
ARCN1	7.17	MDA-MB-468	S2
ARF5	23.91	MDA-MB-468	S2
ARPC3	27.95	MDA-MB-468	S2
ASCC3	18.76	MDA-MB-468	S2
ATP6AP1	21.8	MDA-MB-468	S2
AURKB	2.58	MDA-MB-468	S2
BIRC5	8.95	MDA-MB-468	S2
BLVRA	14.49	MDA-MB-468	S2
BMPR1B	23.51	MDA-MB-468	S2
BRSK1	23.74	MDA-MB-468	S2
C20orf52	22.86	MDA-MB-468	S2
CACNA1G	17.22	MDA-MB-468	S2
CAMKK2	25.13	MDA-MB-468	S2
CARS	24.43	MDA-MB-468	S2
CCNA2	10.65	MDA-MB-468	S2
CCNB3	27.64	MDA-MB-468	S2
CCND2	26.26	MDA-MB-468	S2
CCNF	23.65	MDA-MB-468	S2

CCT2	21.89	MDA-MB-468	S2
CCT7	20.1	MDA-MB-468	S2
CD44	19.22	MDA-MB-468	S2
CD47	6.58	MDA-MB-468	S2
CD68	13.75	MDA-MB-468	S2
CD81	21.54	MDA-MB-468	S2
CD99	24.56	MDA-MB-468	S2
CDC27	9.84	MDA-MB-468	S2
CDC6	26.54	MDA-MB-468	S2
CDC7	15.94	MDA-MB-468	S2
CDH5	6.35	MDA-MB-468	S2
CDKN3	25.39	MDA-MB-468	S2
CFLAR	18.2	MDA-MB-468	S2
CNOT6	12.77	MDA-MB-468	S2
COX4I2	6.82	MDA-MB-468	S2
COX6A1	25.41	MDA-MB-468	S2
CPT1A	19.4	MDA-MB-468	S2
CSE1L	20.96	MDA-MB-468	S2
CTBP1	21.36	MDA-MB-468	S2
CTDP1	26.67	MDA-MB-468	S2
CXXC4	23.07	MDA-MB-468	S2
DAPK2	22.92	MDA-MB-468	S2
DCAMKL1	24.48	MDA-MB-468	S2
DCC	6.31	MDA-MB-468	S2
DDX46	17.34	MDA-MB-468	S2
DDX48	6.09	MDA-MB-468	S2
DHX8	6.96	MDA-MB-468	S2
DKFZp761F	9.73	MDA-MB-468	S2
DSG2	19.65	MDA-MB-468	S2
DTYMK	10.03	MDA-MB-468	S2
DYRK1B	22.94	MDA-MB-468	S2
E2F1	21.84	MDA-MB-468	S2
ECT2	10.87	MDA-MB-468	S2
ENTPD1	25.21	MDA-MB-468	S2
EPB41L2	7.51	MDA-MB-468	S2
EPHB1	15.08	MDA-MB-468	S2
ESRRA	22.6	MDA-MB-468	S2
EVI5L	24.42	MDA-MB-468	S2
EYA3	20.49	MDA-MB-468	S2
FBXL10	11.07	MDA-MB-468	S2
FBXO10	22.98	MDA-MB-468	S2
FBXO5	27.02	MDA-MB-468	S2
FBXW4	25.11	MDA-MB-468	S2
FGR	26.53	MDA-MB-468	S2
FLJ20035	9.03	MDA-MB-468	S2
FLJ30698	11.65	MDA-MB-468	S2
FLJ40852	23.12	MDA-MB-468	S2

FLNA	15.85	MDA-MB-468	S2
FLNB	5.84	MDA-MB-468	S2
FOS	16.73	MDA-MB-468	S2
GALNS	27.6	MDA-MB-468	S2
GBA	24.13	MDA-MB-468	S2
GFPT1	17.45	MDA-MB-468	S2
GMEB2	18.52	MDA-MB-468	S2
GNAI1	24.91	MDA-MB-468	S2
GOT2	8.88	MDA-MB-468	S2
GRAP	5.09	MDA-MB-468	S2
GSG2	4.11	MDA-MB-468	S2
GTF3C1	9.69	MDA-MB-468	S2
GUCY1B3	5.5	MDA-MB-468	S2
hCAP-D3	27.66	MDA-MB-468	S2
HCN3	17.44	MDA-MB-468	S2
HCN4	27.38	MDA-MB-468	S2
HLA-A	25.23	MDA-MB-468	S2
HRAS	16.68	MDA-MB-468	S2
HRMT1L1	20.55	MDA-MB-468	S2
HSD17B4	15.29	MDA-MB-468	S2
HSPB1	27.61	MDA-MB-468	S2
IFT52	21.72	MDA-MB-468	S2
IGF2R	17.64	MDA-MB-468	S2
IGFBP7	27.27	MDA-MB-468	S2
IKBKB	13.41	MDA-MB-468	S2
IMPDH2	11.47	MDA-MB-468	S2
ITGAV	3.82	MDA-MB-468	S2
JAK2	21.69	MDA-MB-468	S2
KCNC3	26.48	MDA-MB-468	S2
KCNC4	11.43	MDA-MB-468	S2
KCNF1	26.64	MDA-MB-468	S2
KCNK15	21.04	MDA-MB-468	S2
KIAA0368	14.87	MDA-MB-468	S2
KIAA1815	22.42	MDA-MB-468	S2
KIF11	6.68	MDA-MB-468	S2
KIF18A	27.63	MDA-MB-468	S2
KIF26B	22.34	MDA-MB-468	S2
KIF2C	28.12	MDA-MB-468	S2
KNTC2	5.35	MDA-MB-468	S2
LRP5	21.2	MDA-MB-468	S2
MAD2L1	20.11	MDA-MB-468	S2
MAP2K4	22.31	MDA-MB-468	S2
MAPK7	24.13	MDA-MB-468	S2
MAPKAPK2	23.23	MDA-MB-468	S2
MAST3	13.81	MDA-MB-468	S2
MEN1	20.73	MDA-MB-468	S2
MIP	26.76	MDA-MB-468	S2

MLL4	17.35	MDA-MB-468	S2
MTAP	26.34	MDA-MB-468	S2
MYD88	15.68	MDA-MB-468	S2
NCOA5	27.65	MDA-MB-468	S2
NET1	24.19	MDA-MB-468	S2
NP	25.14	MDA-MB-468	S2
NR1I3	23.07	MDA-MB-468	S2
OLR1	16.38	MDA-MB-468	S2
OXSM	15.56	MDA-MB-468	S2
PAK2	27.88	MDA-MB-468	S2
PAK7	5.16	MDA-MB-468	S2
PAN3	27.91	MDA-MB-468	S2
PDE4B	18.61	MDA-MB-468	S2
PDE6B	13.01	MDA-MB-468	S2
PDK2	15.48	MDA-MB-468	S2
PHKG1	12.48	MDA-MB-468	S2
PIK4CB	8.89	MDA-MB-468	S2
PIP5K1A	11.37	MDA-MB-468	S2
PIWIL1	23.97	MDA-MB-468	S2
PLCB3	20.22	MDA-MB-468	S2
PLK1	7.83	MDA-MB-468	S2
PLOD3	24.21	MDA-MB-468	S2
POFUT1	23	MDA-MB-468	S2
POLR1D	25.53	MDA-MB-468	S2
POLR2A	1.89	MDA-MB-468	S2
POLR2E	21.45	MDA-MB-468	S2
POLR2F	7.89	MDA-MB-468	S2
POLR2G	9.19	MDA-MB-468	S2
POLR2I	17.08	MDA-MB-468	S2
POLR2L	25.69	MDA-MB-468	S2
PPM1B	19.52	MDA-MB-468	S2
PPM1G	22.43	MDA-MB-468	S2
PPP1CB	14.95	MDA-MB-468	S2
PPP1CC	8.45	MDA-MB-468	S2
PRC1	6.08	MDA-MB-468	S2
PRKAA1	2.43	MDA-MB-468	S2
PRKCD	20.19	MDA-MB-468	S2
PRKCE	27.97	MDA-MB-468	S2
PRKY	16.76	MDA-MB-468	S2
PROCR	13.14	MDA-MB-468	S2
PSMA2	3.84	MDA-MB-468	S2
PSMA3	2.56	MDA-MB-468	S2
PSMA4	1.81	MDA-MB-468	S2
PSMA7	4.22	MDA-MB-468	S2
PSMB1	6.39	MDA-MB-468	S2
PSMB2	9	MDA-MB-468	S2
PSMB3	1.75	MDA-MB-468	S2

PSMB4	3.1	MDA-MB-468	S2
PSMB5	26.93	MDA-MB-468	S2
PSMB6	2.31	MDA-MB-468	S2
PSMB7	19.09	MDA-MB-468	S2
PSMC1	1.32	MDA-MB-468	S2
PSMC2	6.85	MDA-MB-468	S2
PSMC3	8.76	MDA-MB-468	S2
PSMC4	2.52	MDA-MB-468	S2
PSMC5	2.71	MDA-MB-468	S2
PSMC6	3.77	MDA-MB-468	S2
PSMD2	6.46	MDA-MB-468	S2
PSMD7	1.38	MDA-MB-468	S2
PSMD8	3.21	MDA-MB-468	S2
PTEN	12.6	MDA-MB-468	S2
PTK9L	22.16	MDA-MB-468	S2
PTP4A2	24.48	MDA-MB-468	S2
PTPRA	13.68	MDA-MB-468	S2
PTPRK	18.15	MDA-MB-468	S2
PTPRO	27.94	MDA-MB-468	S2
RAD1	13.4	MDA-MB-468	S2
RALBP1	14.49	MDA-MB-468	S2
RARA	26.87	MDA-MB-468	S2
RENT1	24.96	MDA-MB-468	S2
RIOK1	20.85	MDA-MB-468	S2
RIPK4	20.65	MDA-MB-468	S2
RNPC2	4.91	MDA-MB-468	S2
RRM1	8.19	MDA-MB-468	S2
RRM2	20.93	MDA-MB-468	S2
SAE1	27.13	MDA-MB-468	S2
SCAND1	11.21	MDA-MB-468	S2
SCN4A	24.34	MDA-MB-468	S2
SCN7A	15.4	MDA-MB-468	S2
SCYL1	26.31	MDA-MB-468	S2
SLC1A4	15.2	MDA-MB-468	S2
SLC31A1	22.72	MDA-MB-468	S2
SLC39A10	9.68	MDA-MB-468	S2
SLFN12	22.86	MDA-MB-468	S2
SMARCA2	10.24	MDA-MB-468	S2
SMARCA4	18.92	MDA-MB-468	S2
SOD2	27.46	MDA-MB-468	S2
SPAG5	25.37	MDA-MB-468	S2
SRMS	24.69	MDA-MB-468	S2
ST3GAL5	20.4	MDA-MB-468	S2
ST6GALNA(	22.51	MDA-MB-468	S2
STAR	27.33	MDA-MB-468	S2
STK11	23.33	MDA-MB-468	S2
STK32B	15.75	MDA-MB-468	S2

STK40	22.39	MDA-MB-468	S2
STK6	10.02	MDA-MB-468	S2
STMN1	22.87	MDA-MB-468	S2
TACC3	11.39	MDA-MB-468	S2
TCEB3C	4.87	MDA-MB-468	S2
TERF2	18.96	MDA-MB-468	S2
TESK1	26.14	MDA-MB-468	S2
TKT	19.03	MDA-MB-468	S2
TPX2	16.5	MDA-MB-468	S2
TRIM28	7.49	MDA-MB-468	S2
TRPM1	27.8	MDA-MB-468	S2
TSSK4	12.61	MDA-MB-468	S2
TUBA1	9.44	MDA-MB-468	S2
TUBA8	14.66	MDA-MB-468	S2
UBE1DC1	21.85	MDA-MB-468	S2
UBE2C	27	MDA-MB-468	S2
UBE2D4	24.81	MDA-MB-468	S2
UBE2I	21.37	MDA-MB-468	S2
UBR2	23.39	MDA-MB-468	S2
VCP	9.49	MDA-MB-468	S2
ZAK	25.37	MDA-MB-468	S2
ZGPAT	20.65	MDA-MB-468	S2
ZNF335	19.85	MDA-MB-468	S2
ABL2	24.99	ZR-75-1	S2
ACSL4	23.43	ZR-75-1	S2
AK1	16.51	ZR-75-1	S2
AKT2	16.57	ZR-75-1	S2
ANLN	25.09	ZR-75-1	S2
ARCN1	24.22	ZR-75-1	S2
C20orf52	22.9	ZR-75-1	S2
CACNA1F	24.99	ZR-75-1	S2
CD47	17.91	ZR-75-1	S2
CDC2	23.32	ZR-75-1	S2
CFLAR	9.74	ZR-75-1	S2
CNNM4	12.14	ZR-75-1	S2
CPNE1	20.63	ZR-75-1	S2
CRYZ	24.89	ZR-75-1	S2
CSNK1A1	17.65	ZR-75-1	S2
DDX28	18.97	ZR-75-1	S2
DNMT3B	21.13	ZR-75-1	S2
ECT2	24.33	ZR-75-1	S2
ENO2	25	ZR-75-1	S2
FBXO10	13.86	ZR-75-1	S2
FLJ20035	11.93	ZR-75-1	S2
FRAP1	18.35	ZR-75-1	S2
GCLM	11.49	ZR-75-1	S2
GLB1	23.86	ZR-75-1	S2

GUCY1B3	15.36	ZR-75-1	S2
HIPK2	24.12	ZR-75-1	S2
HLCS	23.96	ZR-75-1	S2
HSPA5	25.02	ZR-75-1	S2
HSPB2	12.34	ZR-75-1	S2
IFIH1	21.84	ZR-75-1	S2
IMPDH2	9.24	ZR-75-1	S2
ITPR1	15.4	ZR-75-1	S2
KCNS1	18.49	ZR-75-1	S2
KIF11	15.11	ZR-75-1	S2
KIF2C	22.08	ZR-75-1	S2
KNTC2	21.1	ZR-75-1	S2
LOC34015(	23.34	ZR-75-1	S2
MAP3K13	25.09	ZR-75-1	S2
MAP3K14	15.18	ZR-75-1	S2
MAPKAPK2	13.96	ZR-75-1	S2
MEN1	21.85	ZR-75-1	S2
MINK1	24.81	ZR-75-1	S2
MKI67	21.83	ZR-75-1	S2
MUSK	21.37	ZR-75-1	S2
NBN	20.3	ZR-75-1	S2
NEK7	17.69	ZR-75-1	S2
NEU1	17.38	ZR-75-1	S2
NP	23.54	ZR-75-1	S2
NR1I3	19.07	ZR-75-1	S2
NR3C2	21.7	ZR-75-1	S2
NUAK2	7.9	ZR-75-1	S2
PARP1	9.97	ZR-75-1	S2
PGD	16.92	ZR-75-1	S2
PIP5K1A	24.23	ZR-75-1	S2
PLK3	12.03	ZR-75-1	S2
PLOD3	25.02	ZR-75-1	S2
POLR2A	18	ZR-75-1	S2
PREPL	25.05	ZR-75-1	S2
PSMB3	19.66	ZR-75-1	S2
PTGIR	21.19	ZR-75-1	S2
PTPN22	22.39	ZR-75-1	S2
RAD17	24.83	ZR-75-1	S2
RARA	13.62	ZR-75-1	S2
RPS21	23.72	ZR-75-1	S2
SBK1	24.08	ZR-75-1	S2
SLC25A5	16.14	ZR-75-1	S2
SMPD2	23.19	ZR-75-1	S2
SPAG5	21.86	ZR-75-1	S2
STARD13	20.08	ZR-75-1	S2
STAT1	23.98	ZR-75-1	S2
STK17B	24.53	ZR-75-1	S2

STK25	20.35	ZR-75-1	S2
UROS	19.48	ZR-75-1	S2
VCP	15.06	ZR-75-1	S2
